# Structural Fiber Tract Alterations in Relation to Surgery in Children With a Posterior Fossa Tumor

**DOI:** 10.1002/nbm.70250

**Published:** 2026-02-20

**Authors:** Pien E. J. Jellema, Jannie P. Wijnen, Karina J. Kersbergen, Martijn Froeling, Maarten H. Lequin, Wouter P. Nieuwenhuis, Alberto De Luca, Eelco W. Hoving

**Affiliations:** ^1^ Department of Pediatric Neuro‐Oncology Princess Máxima Center for Pediatric Oncology Utrecht the Netherlands; ^2^ Centre for Image Sciences University Medical Center Utrecht Utrecht the Netherlands; ^3^ Department of Radiology University Medical Center Utrecht Utrecht the Netherlands; ^4^ Edward B. Singleton Department of Radiology Texas Children's Hospital Austin Texas USA; ^5^ Department of Neurology University Medical Center Utrecht Utrecht the Netherlands

**Keywords:** dentato‐rubro‐thalamic tract, diffusion MRI, fiber tractography, intraoperative MRI, pediatric posterior fossa brain tumor

## Abstract

Cerebellar mutism syndrome (CMS) is a potential complication of pediatric posterior fossa tumor (pPFT) resection and may be related to disruption of the dentato‐rubro‐thalamic tract (DRTT). Intraoperative tractography allows assessment of changes to the DRTT during surgery. We evaluated macro‐ and microstructural changes of the DRTT by comparing pre‐ and intraoperative diffusion MRI (dMRI) tractography in patients with pPFT. Pre‐ and intraoperative T1‐weighted and dMRI data were acquired to reconstruct the DRTT, while the arcuate fasciculus (AF) was reconstructed as a control tract. To evaluate macrostructural intraoperative alterations, tract volume and diameter were calculated. Microstructural changes were assessed using fractional anisotropy (FA) and mean diffusivity (MD), both globally and along the tract. Speech disturbances, as a characteristic symptom of CMS, were scored preoperatively and at hospital discharge, based on retrospective neurological reports. In 30 patients (aged > 21 months) with sufficient data quality, intraoperative reductions in tract volumes and diameters were observed (*p* < 0.05) in the decussating DRTT (d‐DRTT) (−45.4% to −10%) and AF (−9.9% to −5.1%), while the non‐decussating DRTT (nd‐DRTT) remained stable. Right d‐DRTT volume loss correlated with ventricular enlargement (r = −0.476, *p* = 0.022). Whole‐tract analyses revealed increased FA in the AF and increased MD in the nd‐DRTT and AF. Along‐tract analyses demonstrated high variance of the MD in the DRTT cerebellar segments. Patients with postoperative speech disturbances showed higher variance of FA and MD along the DRTT, particularly in the left cerebellar segment. pPFT surgery affected the variation of the DRTT microstructure near the resection cavity, whereas the AF remained relatively stable. Although we did not find significant differences in patients with postoperative speech disturbances, we observed a much higher variability in MD in this group, suggesting a potential effect of DRTT disruption in these symptoms.

## Graphical abstract

Children that undergo posterior fossa tumor resection may experience post‐surgery neurological symptoms related to disruption of the dentato‐rubro‐thalamic tract (DRTT). By comparing pre‐ and intraoperative diffusion MRI tractography, we observed more variation in microstructural measures in the DRTT near the resection cavity, which were more pronounced in patients with postoperative speech disturbances.

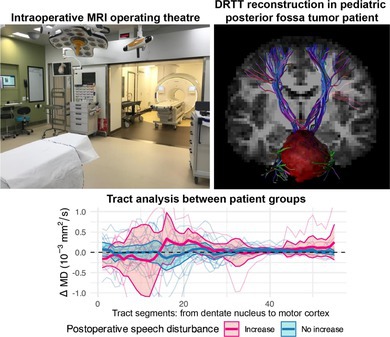

## 1 Introduction

Children with posterior fossa tumors (pPFT) typically undergo resection of the tumor. In some cases, this leads to the onset of cerebellar mutism syndrome (CMS), a postoperative complication that is characterized by speech disturbances [1, 2]. Additional symptoms include ataxia, emotional lability, and cognitive dysfunction [3]. The underlying pathology of CMS has not been elucidated yet, but it may be related to a functional disconnection between the cerebellum and cerebrum, particularly in regions involved in speech and motor control [4]. Given the proximity of the pPFT resection cavity to the dentate nucleus, it is hypothesized that tumor removal may affect the dentato‐rubro‐thalamic tract (DRTT). This fiber tract originates in the dentate nucleus and connects to the cerebral motor cortex via the thalamus. The dentate nucleus integrates a broad range of cerebellar inputs that are related to, among others, language processing, verbal fluency, motor planning, and emotional processing [5, 6, 7, 8, 9]. For all of the above, it has been hypothesized that disruption of the DRTT by pPFT surgery is associated with CMS [10, 11, 12].

Intraoperative MRI (ioMRI) is a highly specialized imaging application where MRI acquisitions are performed during the surgical procedure. It is mainly used to guide immediate further resections when residual tumor tissue is detected [13]. However, it is also a useful tool to monitor structural alterations in healthy brain tissue in response to surgical manipulation [14]. To plan a maximal safe resection, eloquent white matter tracts can be reconstructed preoperatively by means of fiber tractography based on diffusion MRI (dMRI) [15]. However, the accuracy of these reconstructions is compromised due to spatial deformations caused by tumor mass removal and changes in intracranial pressure that induce brain shift [16]. In adults, updating fiber tractography intraoperatively has proven to restore the precision of the reconstructions [17, 18, 19]. Moreover, these intraoperative fiber tract reconstructions can also be compared retrospectively with preoperative data to evaluate morphological changes in white matter tracts during surgery. These changes could result from factors such as the preoperative tumor mass effect, ventricular enlargement, intraoperative brain shift, or postoperative edema due to brain re‐expansion [16].

At the moment, the extent of DRTT structural alterations during pPFT surgery remains poorly characterized, particularly in relation to CMS. Intraoperative fiber tractography offers the potential to capture structural changes to the DRTT as close as possible to the surgical moment. To date, however, intraoperative DRTT tractography has been reported once and only in adult patients [20].

Structural tract changes can be quantified on a macroscopic level by evaluating measures such as tract volume and diameter [21]. On a microscopic level, mean diffusivity (MD) and fractional anisotropy (FA) can provide information on the hindrance of water molecules and their spatial anisotropy for a specific tract. Higher FA values indicate more coherent diffusion along organized fiber bundles. Lower MD values reflect reduced extracellular space or tissue water content. The combination of high FA and low MD can be interpreted as greater fiber integrity [22, 23, 24, 25, 26]. Integrating macro‐ and microstructural analyses could provide a comprehensive understanding of how surgical manipulation affects the DRTT and may clarify the relationship between structural alterations and postoperative neurological complications.

In our earlier work, we demonstrated a tractography methodology capable of producing reliable intraoperative DRTT reconstructions for pPFT surgery, despite the inherently lower signal‐to‐noise ratio (SNR) of ioMRI acquisitions [27]. Building on this work, the present study aims to investigate the effect of pPFT surgery on the DRTT by analyzing its macro‐ and microstructural changes between preoperative and intraoperative measurements as compared with a distant control tract.

We hypothesize that the DRTT undergoes greater macrostructural and microstructural changes than control fiber tracts distant from the resection cavity. In addition, we hypothesize that larger tumor and ventricular volumes are associated with more pronounced macrostructural alterations. Finally, we expect that microstructural changes in the DRTT are particularly evident in patients who develop postoperative neurological complications.

## 2 Methods

### 2.1 Study Population

Between January 2023 and June 2024, 39 patients with pPFT aged between 15 months and 17 years (17 females) were consecutively included from the neurosurgical department of the Princess Máxima Centre for Pediatric Oncology, Utrecht, the Netherlands. The local ethics committee approved this study (PMCLAB2022.0379). All subjects and/or caregivers provided written informed consent. In addition to demographic information on age and sex assigned at birth, we collected clinical data on tumor type, location, and midline asymmetry. Surgical characteristics included extraventricular drain placement, history of previous resection, extent of resection, additional therapies, and mortality. These variables were collected for descriptive purposes and assessment of potential confounding.

### 2.2 Experimental Setup

**FIGURE 1:**
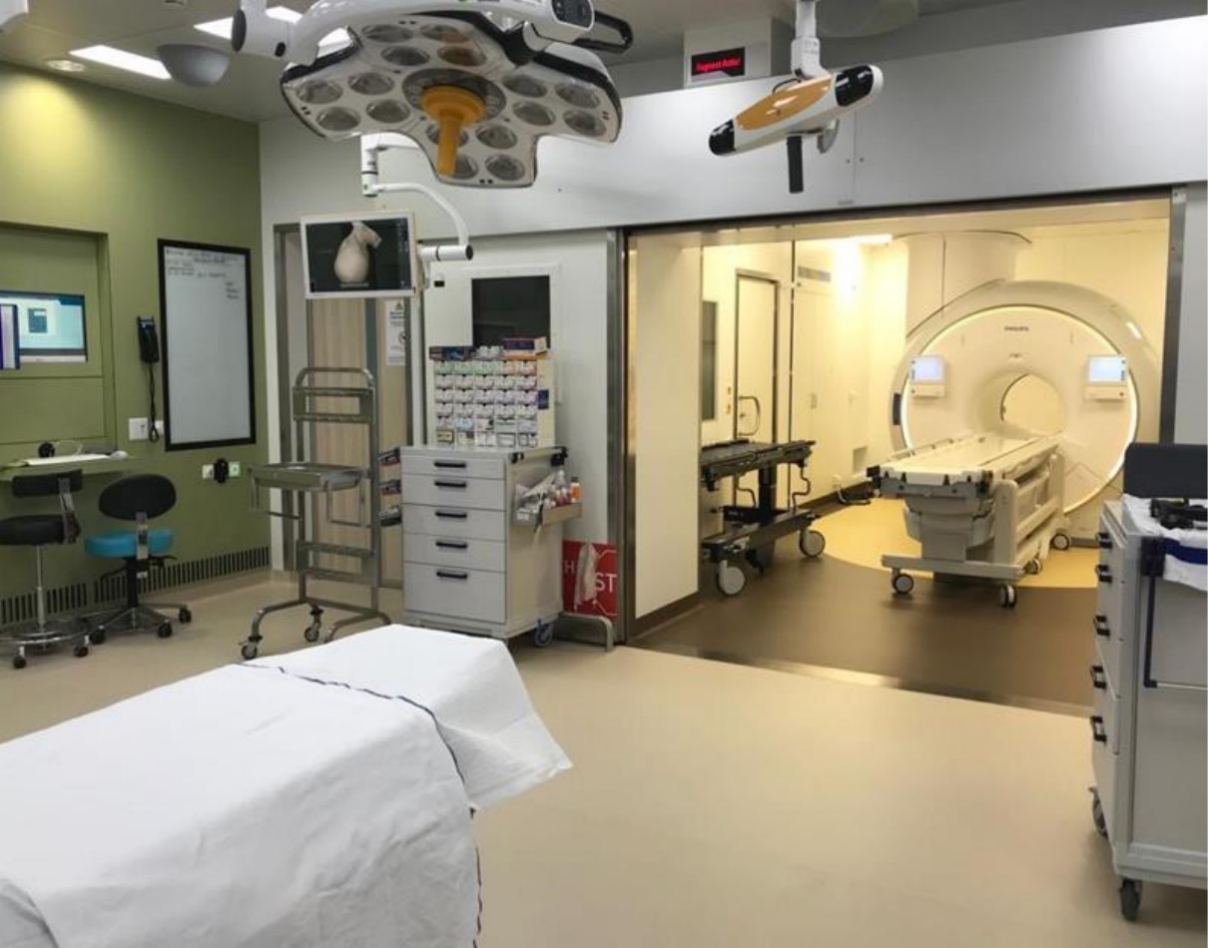
Set up of the intraoperative MRI operating theatre.

For this study, MRI data of all included patients were obtained at two time points. Preoperative scans were obtained before the craniotomy. Intraoperative scans were acquired after resecting the majority of the tumor mass, with an open skull, when the neurosurgeon decided to check for residual tumor tissue. If residual tumor tissue was detected on the intraoperative scan, the neurosurgeon could continue the resection. All MR images were acquired on a 3 Tesla intraoperative MRI scanner (Philips Ingenia ElitionX MR‐OR system, 70 cm bore, Philips Healthcare, Best, The Netherlands, Figure 1) with a similar setup as previously described [27]. In short, the setup comprised two RF coils, with the patient positioned prone and the head secured in a head clamp. For intraoperative scans, the resection cavity was filled with 0.9% NaCL solution, covered with dura and cottonoids, and the skin was temporarily approximated with a few temporary stitches to reduce susceptibility artefacts.

### 2.3 Imaging Protocol

For each patient and at each time point, a T1‐weighted (T1w) scan for anatomical reference and multi‐shell diffusion MRI (dMRI) was acquired. The dMRI acquisition consisted of a total of six non‐diffusion‐weighted images (b = 0 s/mm^2^, referred to as b0), 20 diffusion‐weighted images at a b = 1000 s/mm^2^, and 32 diffusion‐weighted images at a b = 2000 s/mm^2^. Full protocol details have been described previously [27]. The total dMRI scan time was approximately 9 min and was optimized to meet the constraints of the intraoperative setting [28].

### 2.4 Whole‐Brain Fiber Tractography

In preparation for tract segmentation, we performed whole‐brain fiber tractography analysis. The dMRI data were preprocessed following the previously described approach [27, 29, 30, 31, 32, 33, 34, 35, 36, 37, 38] to correct for artifacts. To reconstruct fiber orientation distributions (FOD) in white matter, the Generalized Richardson Lucy spherical deconvolution method [29] was applied while accounting for partial volume effects from gray matter and cerebrospinal fluid (CSF). This step allows us to capture multiple fiber orientations within each voxel [39].

Intraoperative dMRI data have inherently lower SNR than other research‐oriented protocols. Tractography parameters were, therefore, based on settings previously optimized for this type of data [27]. We performed deterministic tractography using FACT propagation (ExploreDTI [30]). Seeds were placed on a 1‐mm isotropic grid throughout the whole‐brain mask. Propagation used a 1‐mm step size and terminated when FOD amplitude < 0.01 or when the turn angle exceeded 60° between successive steps. The gray‐white matter interface was also used as a streamline termination criterion.

### 2.5 Tract Segmentation

To accurately segment the DRTT from the whole brain tractography data, anatomically identified regions of interest (ROI) were used to define its expected trajectory [40, 41, 42]. These ROI gates then served as inclusion (AND) or exclusion (NOT) waypoints for tractography. Inclusion ROIs comprised the dentate nucleus, thalamus, and motor cortex region. The motor cortex ROI combined the primary motor cortex, supplementary motor area, and premotor cortex. This combined motor cortex ROI provided a more comprehensive representation of the motor output of the DRTT, consistent with prior reports relating these areas to motor speech initiation [40, 42]. Exclusion ROIs included the corpus callosum and tumor mask.

ROI segmentation combined automated atlas‐based methods with manual adjustments to account for tumor‐related deformation and intraoperative brain shift. ROIs were segmented separately on the pre‐ and intraoperative datasets and were initially segmented on T1w images, which provided better contrast and higher resolution, before registration to the native dMRI space for tractography analyses. Whole brain white matter, gray matter, thalamus, and ventricular masks were segmented using the Synthseg convolutional neural network [43, 44]. The dentate nucleus was retrieved from the SUIT cerebellar atlas Diedrichsen et al. [45], and the tumor mask was semi‐manually segmented using the Smart Brush tool in the Brainlab neuronavigation system (Smart Brush, Brainlab, Munich, Germany). The combined motor cortex ROI [46] and the corpus callosum [47] were directly registered to the native space. A pediatric neuroradiologist (W.N., 5 years of experience) verified the anatomical accuracy of the dentate nucleus and tumor volume using both T1w and T2‐weighted (T2w) scans available in the patient archiving system.

Two components of the DRTT were reconstructed: the decussating DRTT (d‐DRTT) and non‐decussating DRTT (nd‐DRTT). The d‐DRTT decussates in the brainstem and connects the dentate nucleus with the contralateral motor cortex, whereas the nd‐DRTT does not and connects the dentate nucleus with the ipsilateral motor cortex. Their anatomical trajectories have been previously described [27, 41, 48, 49, 50, 51, 52].

In addition to the DRTT, we segmented the arcuate fasciculus (AF), as a control tract located anatomically distant from the pPFT resection cavity. The AF is used to evaluate the consistency of measurements and the presence of systematic offsets unrelated to the surgical procedure between the preoperative and intraoperative scans. The AF was segmented from the whole brain tractography using the white matter analysis pipeline [53]. Because of the permissive tractography parameters required for reliable DRTT reconstruction in the low SNR intraoperative data, additional filtering was applied to the AF to remove short spurious streamlines. To this end, the median length of the AF was calculated for each subject and normalized by individual brain height (calculated in the feet‐head direction as a proxy of brain size). Then, streamlines of the individual AFs were excluded if they were shorter than the first percentile of the normalized median AF distribution.

### 2.6 Macrostructural Metrics

Macrostructural changes of the brain were quantified at two levels: global brain tissue volumes and tract‐specific volumes. Global brain tissue volumes included supratentorial white matter, gray matter, ventricles, and tumor. The ventricle volume was normalized to the total volume of healthy brain tissue (supratentorial white matter, gray matter, and ventricles). Preoperative tumor volume was normalized to the combined volume of the tumor and healthy brain tissue, the latter defined as supratentorial white matter, gray matter, and ventricles.

Tract volume was calculated as the count of voxels that contain at least one streamline and normalized by the total supratentorial white matter volume. Infratentorial white matter volume was not used for normalization as it is more prone to inaccuracy given the location of the pPFT resection cavity. Additionally, for the reconstructed tracts, we calculated the diameter as follows: $\text{diameter}\;\left(\mathrm{mm}\right)=2\sqrt{\frac{\text{volume}}{\pi \times \text{length}}}$ [21].

### 2.7 Microstructural Tract Metrics

To quantify potential microstructural differences between pre‐ and intraoperative measurements, two conventional and complementary metrics from diffusion tensor imaging were used: FA and MD. Microstructural tract values were estimated using two approaches: average values across each tract and values sampled along the tract. For both methods, the absolute and relative changes between pre‐ and intraoperative measurements were calculated. The whole‐tract FA and MD values were obtained by calculating the average values across streamlines [54]. For the along‐tract analyses, the average centerline was first calculated. The centerline was subdivided into 55 equally spaced segments, based on the average streamline length (≈138 mm) and voxel size (=2.55 mm) [55]. Then, the points of all streamlines were projected to the closest centerline segment, and their mean values were calculated. For the DRTT, the numbering of the tract segments is progressive according to their trajectory from the dentate nucleus to the motor cortex. Similarly, segments of the AF are progressively numbered based on their positioning from Wernicke's to Broca's area.

### 2.8 SNR

The SNR was calculated as the mean of the signal intensity of each voxel of the white matter mask of all b0 images, divided by their standard deviation. The average SNR was calculated over all voxels located within the white matter mask.

### 2.9 Neurology

To capture postoperative neurological changes, we incorporated the speech disturbance subscore of the Scale for the Assessment and Rating of Ataxia (SARA [56]) assessed by a trained pediatric neurologist (K.J.K. with 5 years of experience). As part of routine clinical care in our center, the SARA is assessed before surgery (T0), before hospital discharge after surgery (T1), 3 months after surgery (T2), and 1 year after surgery (T3). This data was retrieved retrospectively from the neurological report in the patient archive system. Speech was rated during normal conversation with the pPFT patient from 0 (*normal*) to 1 (*suggestion of speech disturbance*), 2 (*impaired speech, but easy to understand*), 3 (*occasional words difficult to understand*), 4 (*many words difficult to understand*), 5 (*only single words understandable*) to 6 (*speech unintelligible/anarthria*). To explore the potentially impaired functionality of the DRTT after surgery, we included a categorized measure of speech disturbance, which was defined as an increase of at least one point on the SARA subscale at the early postoperative assessment (T1) relative to the presurgical assessment (T0).

### 2.10 Statistical Comparison of Macro‐ and Microstructure

Statistical analyses were performed to assess (1) within‐patient structural changes between preoperative and intraoperative measurements and (2) differences in tract alterations between patients with and without postoperative speech disturbances.

#### 2.10.1 Within‐Patient Analyses

Within patients, differences between preoperative and intraoperative measurements of supratentorial white matter, gray matter, and ventricular volumes were assessed using the Wilcoxon signed‐rank test for paired data. Likewise, tract volume, diameter, whole‐tract FA, and MD were analyzed across time points using linear mixed‐effects models with patients as a random effect and subsequent correction for either SNR or age. For all macrostructural and whole‐tract microstructural metrics, effect sizes (Cohen's *d*) were calculated. Effect sizes were defined as negligible (0–0.2), small (0.2–0.5), moderate (0.5–0.8), and large (> 0.8). To explore potential confounding, correlations were performed to determine whether changes in tract volume or diameter were related to ventricular or tumor volume.

#### 2.10.2 Between‐Patient Analyses

Between‐group comparisons tested whether whole‐tract and along‐tract microstructural changes differed between patients with and without postoperative speech disturbances, using the Wilcoxon rank‐sum test. Logistic regression was applied to evaluate associations of age and sex assigned at birth with the presence of postoperative speech disturbances. Along‐tract analyses of the group mean ΔFA and ΔMD employed z‐tests to assess whether mean changes at each tract segment differed from zero. All statistical tests were corrected for multiple comparisons using the Benjamini–Hochberg false discovery rate procedure, and statistical significance was defined as *p* < 0.05.

## 3 Results

### 3.1 Patient Characteristics

Of the 39 patients with pPFT who underwent ioMRI acquisition during their surgical procedure, nine children were excluded due to age below 21 months or due to poor data quality at one of the two acquisitions. The 30 remaining children (14 females) had an average age of 7.6 ± 4.6 years. Tumor diagnoses of pilocytic astrocytoma (40%), medulloblastoma (27%), and ependymoma (20%) were most prevalent in our population (Table 1). Tumors were mostly located symmetrically between the hemispheres (73%) and specifically in the fourth ventricle (53%), brain stem (13.3%), or vermis (13.3%). Before the tumor resection surgery, in 53% of the children, an extra ventricular drain had been placed, and 13% had received previous resection. In 73% of all surgeries, a complete extent of resection had been reported by the neurosurgical team, which was followed up by either radiotherapy (23%), chemotherapy (17%), or both (13%). Two patients in our study population passed away.

**TABLE 1:** Demographic and clinical characteristics of the patient population.

*Characteristic*	*N* = 30^a^
Sex assigned at birth	
Female	14 (47%)
Male	16 (53%)
Age (years)	7.6 (4.6)
Tumor type	
Pilocytic astrocytoma	12 (40%)
Medulloblastoma	8 (27%)
Ependymoma	6 (20%)
Atypical teratoid rhabdoid tumor	1 (3.3%)
BCOR Sarcoma	1 (3.3%)
Choroid plexus papilloma	1 (3.3%)
Ganglioglioma	1 (3.3%)
Tumor location	
4th ventricle	17 (56.7%)
Brain stem	4 (13.3%)
Vermis	4 (13.3%)
Cerebellar hemisphere	3 (10%)
Pinealis	1 (3.3%)
Cerebellopontine angle	1 (3.3%)
Asymmetry to midline	
Left	3 (10%)
Right	5 (17%)
Symmetric	22 (73%)
Extra ventricular drain inserted	16 (53%)
Previous resection	4 (13%)
Extent of resection	
Complete	22 (73%)
Partial	8 (27%)
Additional therapy	
Radiotherapy	7 (23%)
Chemotherapy	5 (17%)
Radio‐ and chemotherapy	4 (13%)
No treatment	14 (47%)
Mortality	2 (6.7%)

^a^ *n* (%); Mean (SD).

At the early postoperative speech disturbance assessment (T1), 10 patients showed an increase in symptoms, while 18 patients showed no increase. At follow‐up, the number of patients with speech disturbance symptoms decreased to six at T2 and five at T3, as expected for CMS recovery. Neurological data were missing for two patients: one passed away before the postoperative assessment, and one had incomplete data. Logistic regression indicated that age and sex assigned at birth did not significantly predict increased postoperative speech disturbances (*p* > 0.05).

### 3.2 Generic Brain Morphology

Overall brain morphology within patients remained stable between preoperative and intraoperative acquisitions (Table 2). We found that supratentorial white matter, gray matter, and ventricle volumes did not differ significantly between the pre‐ and intraoperative measurements (*p* > 0.05). Normalized preoperative tumor volume was on average 3.5% ± 2.7% of the combined volume of the tumor and healthy brain tissue. However, mean white matter SNR decreased significantly by 9.4% from the preoperative to intraoperative acquisition (*p* = 0.004).

**TABLE 2:** Brain and tumor morphology characteristics. Tumor volume is normalized to supratentorial white matter volume. *p* values are retrieved from Wilcoxon signed‐rank tests for non‐parametric paired data. Bold *p* values are significant (*p* < 0.05).

Characteristic	Preoperative*	Intraoperative*	*p*
White matter volume (cm^3^)	386.60 (55.19)	384.29 (59.15)	0.843
Grey matter volume (cm^3^)	560.03 (46.60)	551.94 (52.12)	0.449
Ventricle volume (%)	6.11 (4.21)	6.06 (3.86)	0.947
Tumor volume (%)	3.47 (2.68)	—	NA
Mean white matter SNR	24.36 (4.60)	22.07 (3.96)	0.004

* Mean (SD).

### 3.3 Macrostructural Tract Changes

**FIGURE 2:**
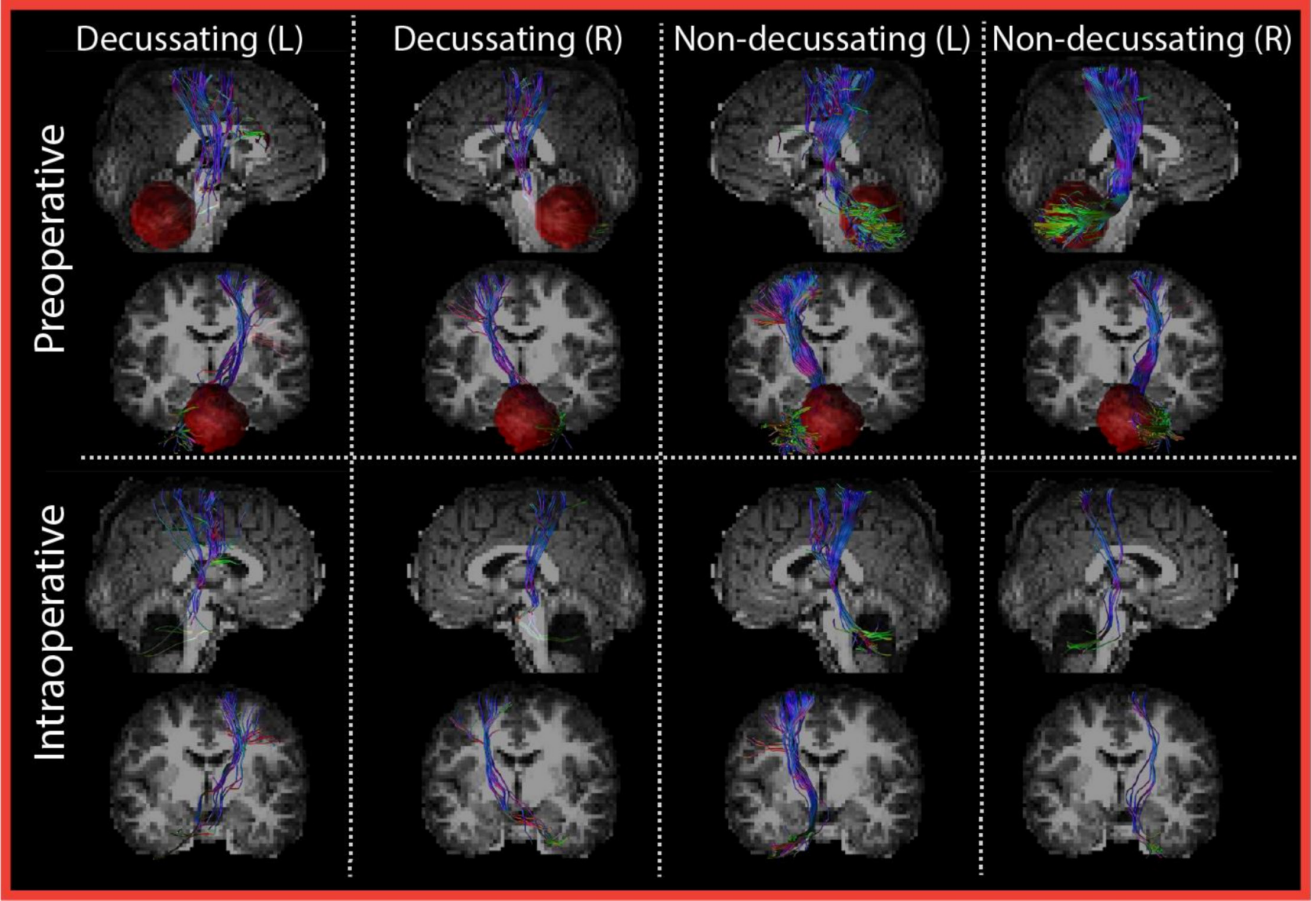
Dentato‐rubro‐thalamic tract (DRTT) reconstructions. Examples of pre‐ and intraoperative DRTT reconstructions of Patient 38. The first two columns show the bilateral decussating DRTTs (d‐DRTT) that connect the dentate nucleus to the contralateral motor cortex, while the last two columns show non‐decussating DRTTs (nd‐DRTT) connecting the dentate nucleus with the ipsilateral motor cortex. Left (L) or right (R) d‐DRTT/nd‐DRTT refers to the cerebellar hemisphere of the dentate nucleus from which the tract originates. Preoperative tumor volume is indicated in red.

**FIGURE 3:**
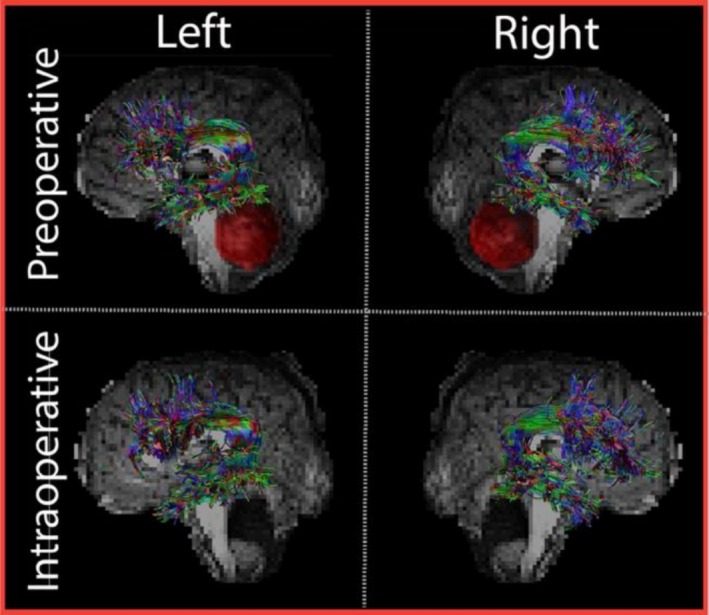
Arcuate fasciculus (AF) reconstructions. Examples of pre‐ and intraoperative AF reconstructions of Patient 38 in the left and right hemispheres. Preoperative tumor volume is indicated in red.

**FIGURE 4:**
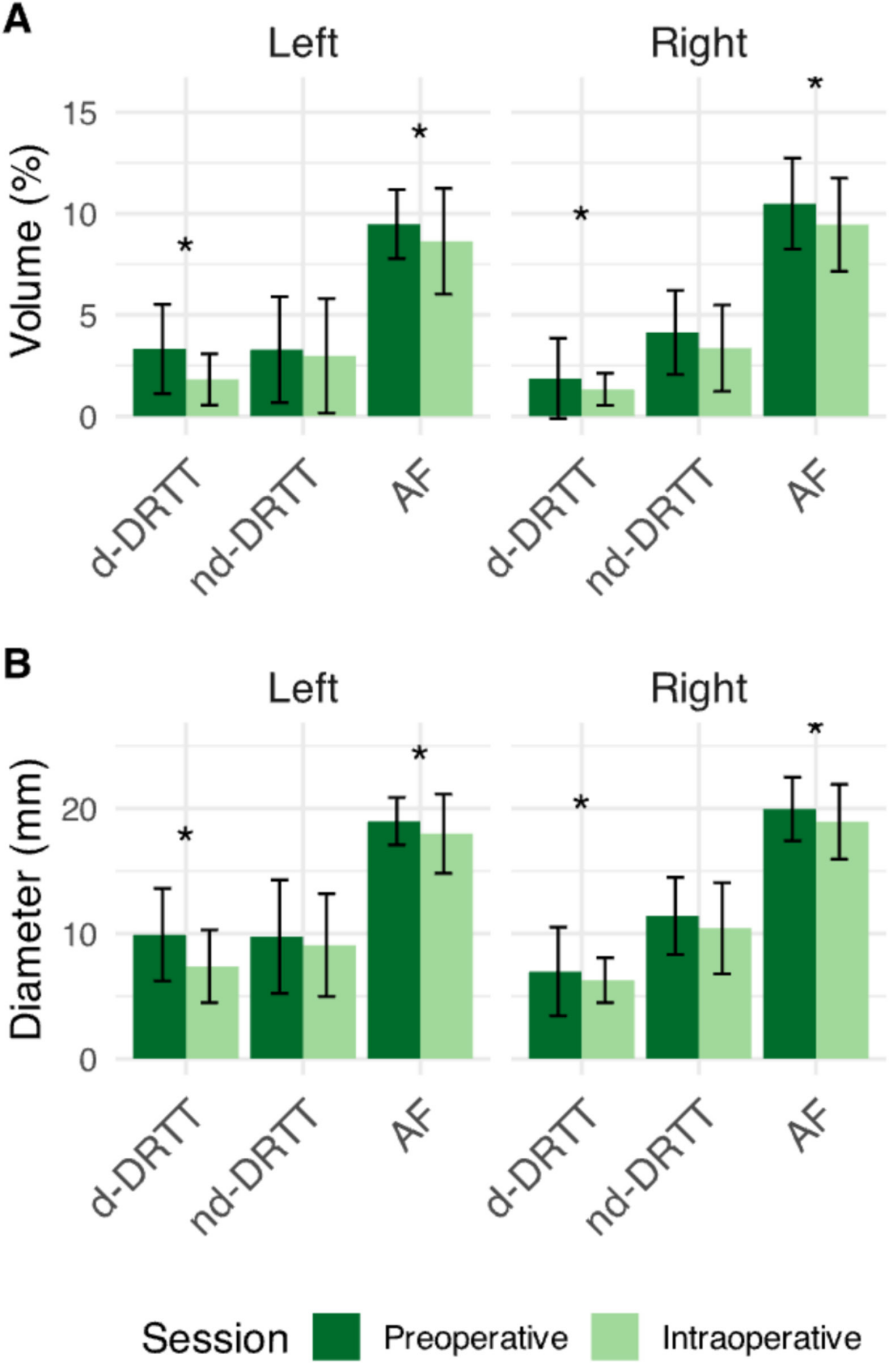
Macrostructural tract changes. Mean tract volume (A) and diameter (B) with standard deviation error bars are shown for the decussating and non‐decussating dentato‐rubro‐thalamic tracts (d‐DRTT or nd‐DRTT) and arcuate fasciculus (AF). Tract volumes are expressed as percentages of supratentorial white matter volume. Preoperative data (dark green) were acquired before craniotomy, and intraoperative data (light green) were acquired with an open skull to evaluate potential signs of residual tumor on MRI. Left or right d‐DRTT/nd‐DRTT corresponds to the cerebellar hemisphere of the dentate nucleus where the tract originates. Asterisks (*) indicate a significant difference (*p* < 0.05) between pre‐ and intraoperative measures, assessed using a linear mixed‐effects model within patients.

Most AF reconstructions and DRTT reconstructions were successfully reconstructed (Data S1 and S2). However, one AF (preoperative), 12 d‐DRTTs (four preoperative and eight intraoperative), and six nd‐DRTTs (one preoperative, five intraoperative) failed to reconstruct on either the left or the right side. Figures 2 and 3 display examples of the DRTT and AF reconstructions of the same patient. After evaluation within patients of macrostructural changes in tract volume and diameter between the pre‐ and intraoperative measurements, we found significant intraoperative reductions for the d‐DRTT and AF, whereas the nd‐DRTT remained stable. These effects remained significant after correction for multiple comparisons, as shown in Figure 4. Specifically for the d‐DRTT, volume decreased by 45.4% on the left side (*p* = 0.011, d = 0.83 [large]) and 28.6% on the right side (*p* = 0.011, d = 0.35 [small]). Correspondingly, the diameter of the d‐DRTT decreased by 25.4% on the left side (*p* = 0.031, d = 0.76 [moderate]) and 10% on the right side (*p* = 0.031, d = 0.24 [small]). For the AF, smaller decreases were observed than for the d‐DRTT, with volume decreasing by 8.8% on the left side (*p* = 0.009, d = 0.38 [small]) and 9.9% on the right side (*p* = 0.009, d = 0.47 [small—moderate]). The diameter of the AF decreased by 5.3% on the left side (*p* = 0.009, d = 0.38 [small]) and 5.1% on the right side (*p* = 0.009, d = 0.37 [small]). No intraoperative macrostructural changes were observed in the nd‐DRTT. Volume and diameter remained stable on both sides (*p* > 0.05), with negligible effect sizes on the left and small effects on the right (volume: d = 0.37; diameter: d = 0.29). Correction for age did not alter the significance of the changes in these macrostructural metrics. After correcting for SNR, the effects remained significant but at higher *p*‐values for d‐DRTT volume (left and right: *p* = 0.038) and diameter (left and right: *p* = 0.047). Similarly, for the AF, significance remained but with higher *p* values for both volume (left and right: *p* = 0.047) and diameter (left and right: *p* = 0.038) after correction for SNR.

For the tracts that showed statistically significant decreases in macrostructural metrics, a correlation analysis was conducted with tumor or ventricle volume. This analysis revealed a statistically significant negative correlation in the right d‐DRTT between tract volume reduction and larger ventricular volumes (r = −0.476, *p* = 0.022). No other significant correlations with tract volume or diameter reduction in the left d‐DRTT or AF were found with tumor or ventricular volume.

### 3.4 Whole‐Tract Microstructural Metrics

#### 3.4.1 Within‐Patient Metrics

**FIGURE 5:**
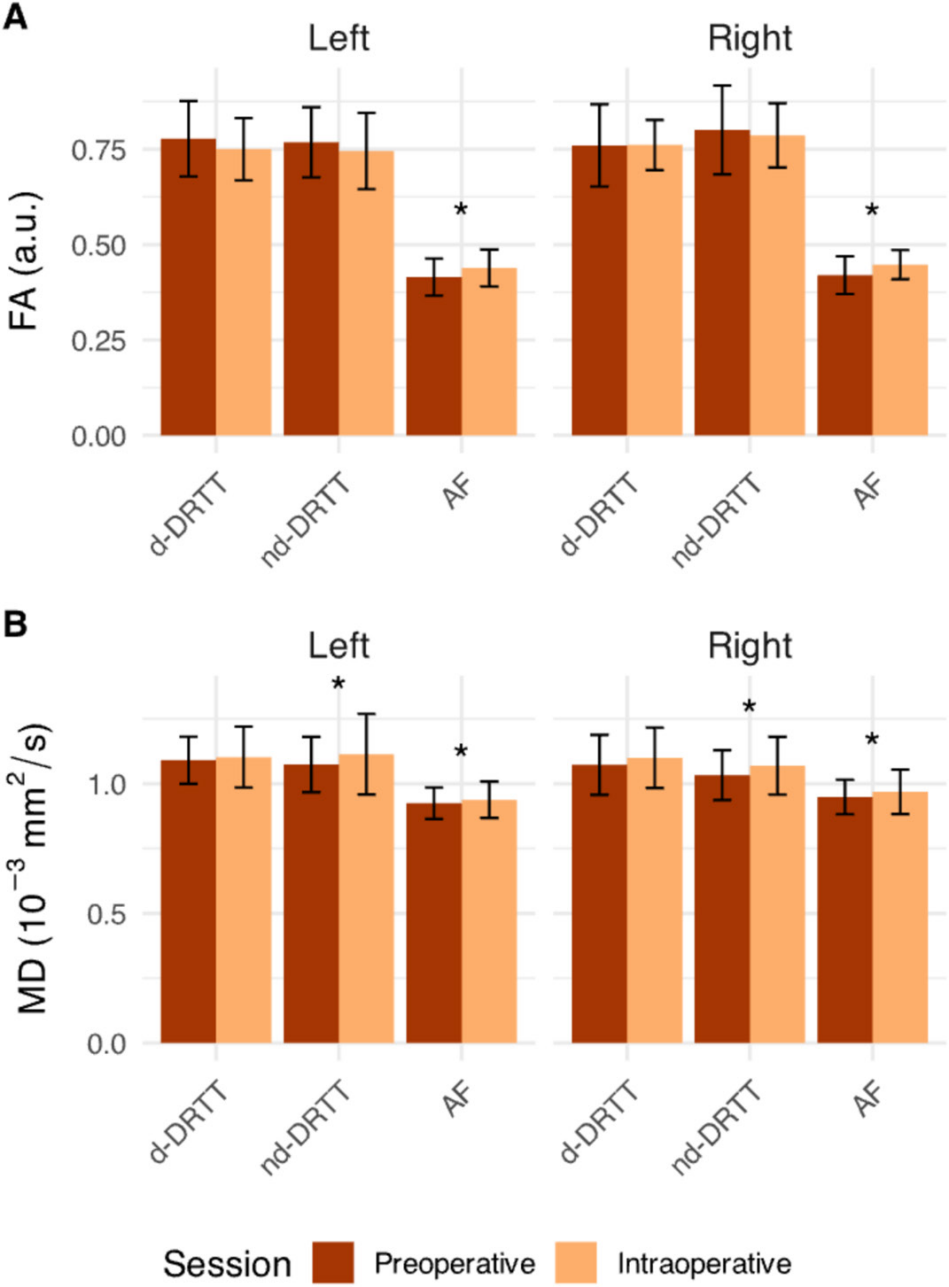
Whole‐tract microstructural tract changes. Whole‐tract mean fractional anisotropy (FA, A) and mean diffusivity (MD, B) with standard deviation error bars are shown for the decussating and non‐decussating dentato‐rubro‐thalamic tracts (d‐DRTT or nd‐DRTT) and arcuate fasciculus (AF). Preoperative data (dark orange) were acquired before craniotomy, and intraoperative data (light orange) were acquired with an open skull to evaluate potential signs of residual tumor on MRI. Left or right d‐DRTT/nd‐DRTT corresponds to the cerebellar hemisphere of the dentate nucleus where the tract originates. Asterisks (*) indicate a significant difference (*p* < 0.05) between pre‐ and intraoperative measures, assessed using a linear mixed‐effects model within patients.

The evaluation of whole‐tract microstructural changes between pre‐ and intraoperative measurements is shown in Figure 5. For FA, significant increases in whole‐tract measurements were observed for the AF. Specifically, the left AF increased with 5.9% (*p* < 0.001, d = 0.49 [small–moderate]) and the right AF with 7.1% (*p* < 0.001, d = 0.62 [moderate]). No significant FA changes were observed in either component of the DRTT (*p* > 0.05), though small effects of decrease were observed on the left for the d‐DRTT (d = 0.30) and nd‐DRTT (d = 0.23), and negligible effects on the right (d < 0.2). For MD, assessment of whole‐tract alterations did show significant increases for the nd‐DRTT and AF. The left nd‐DRTT increased with 3.5% (*p* = 0.049, d = 0.30 [small]) and the right with 3.8% (*p* = 0.049, d = 0.35 [small]). For the MD of the AF, we observed smaller increases than for the nd‐DRTT, namely 1.5% on the left side (*p* = 0.049, d = −0.20 [negligible]) and 2.0% on the right side (*p* = 0.049, d = 0.25 [small]). The d‐DRTT mean MD increase was not significant (*p* > 0.05), with a negligible effect on the left (d < 0.2) and small on the right (d = −0.24). Correction for age did not alter the significance of the changes in whole‐tract mean FA between pre‐ and intraoperative measurements. After adjusting for age, differences in mean MD were not significant (*p* > 0.05) for any tract. Similar results were observed when SNR was included as a covariate instead of age.

#### 3.4.2 Between‐Patient Metrics

**FIGURE 6:**
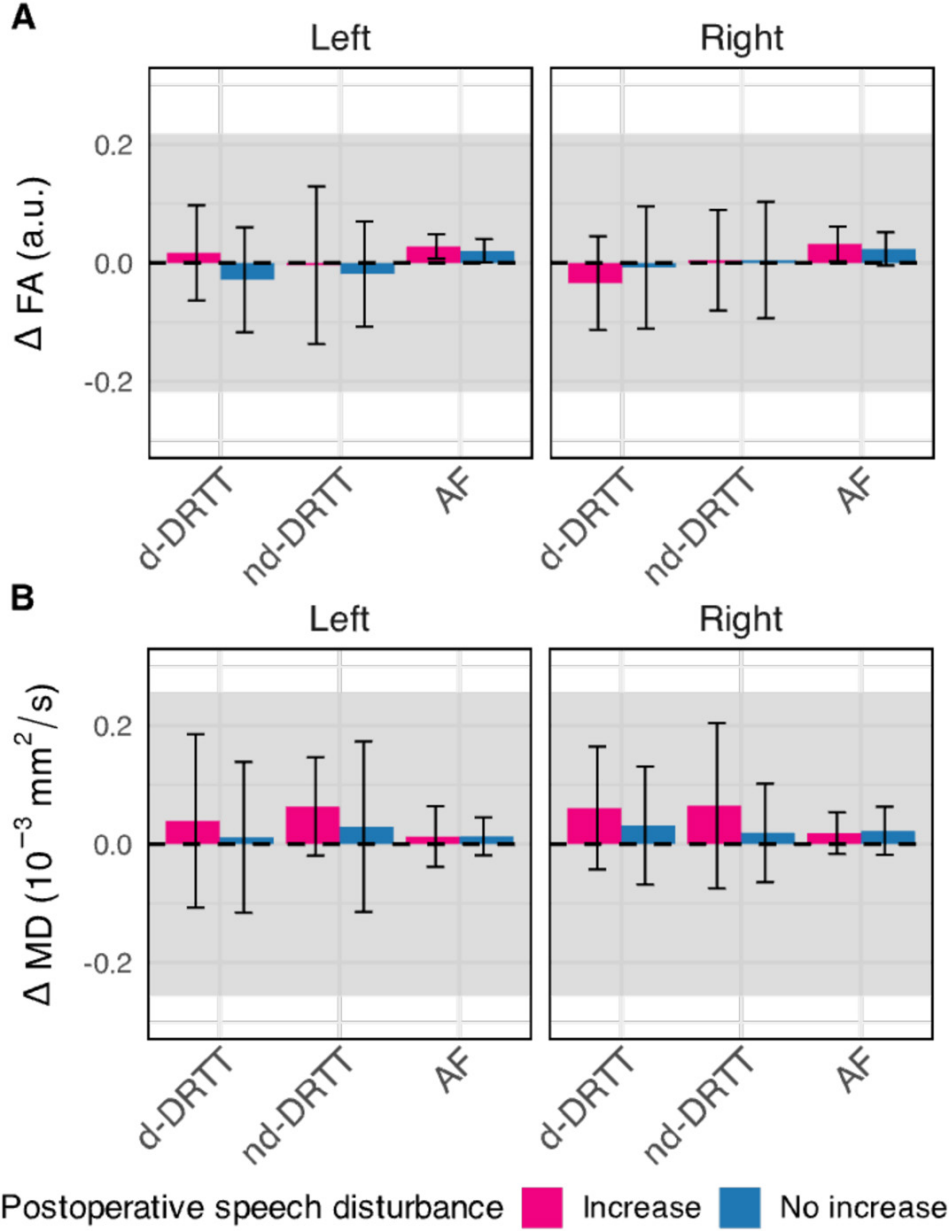
Change in whole‐tract microstructure between patients with and without postoperative speech disturbance. (A) Difference in fractional anisotropy (FA) and (B) in mean diffusivity (MD) between intraoperative and preoperative data, evaluated in patients with (pink) and without (blue) an increase in postoperative speech disturbance symptoms. Bars represent mean change values (Δ) with standard deviation error bars for the decussating and non‐decussating dentato‐rubro‐thalamic tracts (d‐DRTT, nd‐DRTT) and the arcuate fasciculus (AF). Left or right d‐DRTT/nd‐DRTT corresponds to the cerebellar hemisphere of the dentate nucleus where the tract originates. Shaded areas denote the group‐averaged standard deviation of FA or MD; the dashed line marks Δ = 0. No significant differences (*p* > 0.05, Wilcoxon rank‐sum test) were observed between groups.

Figure 6 shows the intraoperative changes of whole‐tract FA or MD for patients with pPFT with or without increased postoperative speech disturbances. Across all tracts and sides, no significant differences were observed between patient groups with small to moderate effect sizes (all *p* > 0.05). None of these intraoperative changes in FA or MD exceeded the group‐averaged standard deviation.

### 3.5 Along‐Tract Microstructural Metrics

#### 3.5.1 Within‐Patient Metrics

**FIGURE 7:**
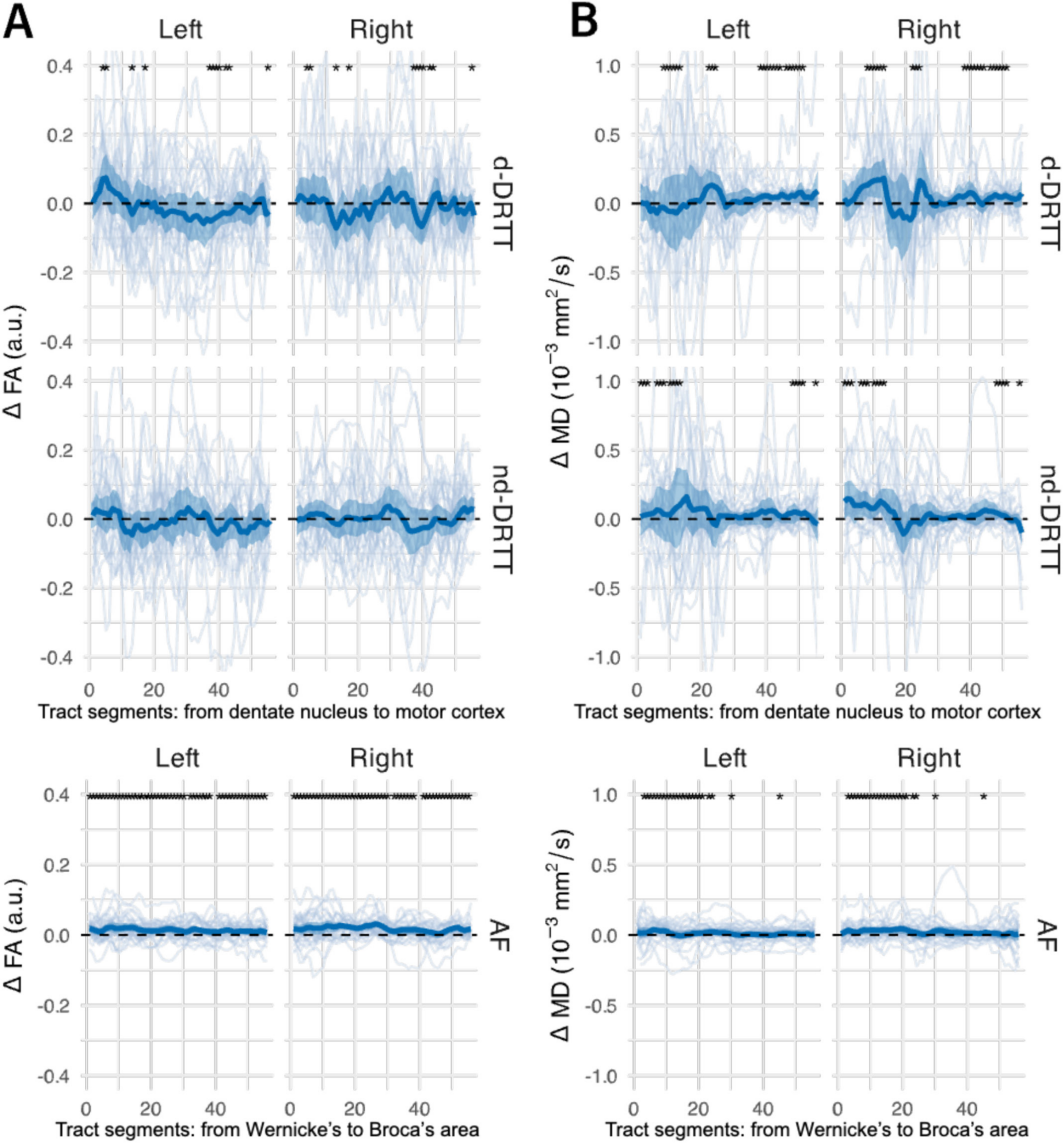
Microstructural changes along the tracts. Fractional anisotropy (FA; A) changes between intraoperative and preoperative measurements along the decussating dentato‐rubro‐thalamic tract (d‐DRTT), non‐decussating DRTT (nd‐DRTT), and arcuate fasciculus (AF). Changes in mean diffusivity (MD; B) are shown for the DRTT and AF. “Left” and “Right” indicate the cerebellar hemisphere of the dentate nucleus where each DRTT originates. Thick lines represent group means, shaded areas indicate 95% confidence intervals, thin lines show individual patient trajectories, and the dashed line marks Δ = 0. Asterisks (*) mark tract segments with a significant non‐zero change (z‐test, *p* < 0.05).

An analysis of microstructural differences along the tracts between pre‐ and intraoperative data revealed high variance in FA for both the d‐DRTT and nd‐DRTT on both sides across the whole patient group, as shown in Figure 7A. In comparison, the AF displayed considerably lower variance in FA along its trajectory. AF segments showed more often significant non‐zero differences (left: 81.8%, right: 74.5%) compared with the d‐DRTT (left: 14.5%, right: 10.9%), likely because of the high variance observed for the DRTT. However, the group‐averaged absolute maximum ΔFA was larger for the d‐DRTT (left: 0.07, right: 0.07) and nd‐DRTT (left: 0.05, right: 0.04) than for the AF (left: 0.02, right: 0.03). For MD, along‐tract analysis across the whole patient group revealed greater variability in the d‐DRTT and nd‐DRTT near the dentate nucleus compared with their projections to the motor cortex, as shown in Figure 7B. In comparison, the AF demonstrated little variance in MD along its trajectory. The proportion of segments with MD significantly different from zero varied by side: on the left, d‐DRTT (27.3%) exceeded AF (12.7%) while nd‐DRTT was lower (5.5%); on the right, AF (34.5%) exceeded both d‐DRTT (23.6%) and nd‐DRTT (21.8%). Group‐averaged absolute maximum ΔMD (scaled to 10^3^) was larger for the d‐DRTT (left: 0.13, right: 0.18) and nd‐DRTT (left: 0.16, right: 0.15) than for the AF (left: 0.04, right: 0.04).

#### 3.5.2 Between‐Patient Metrics

**FIGURE 8:**
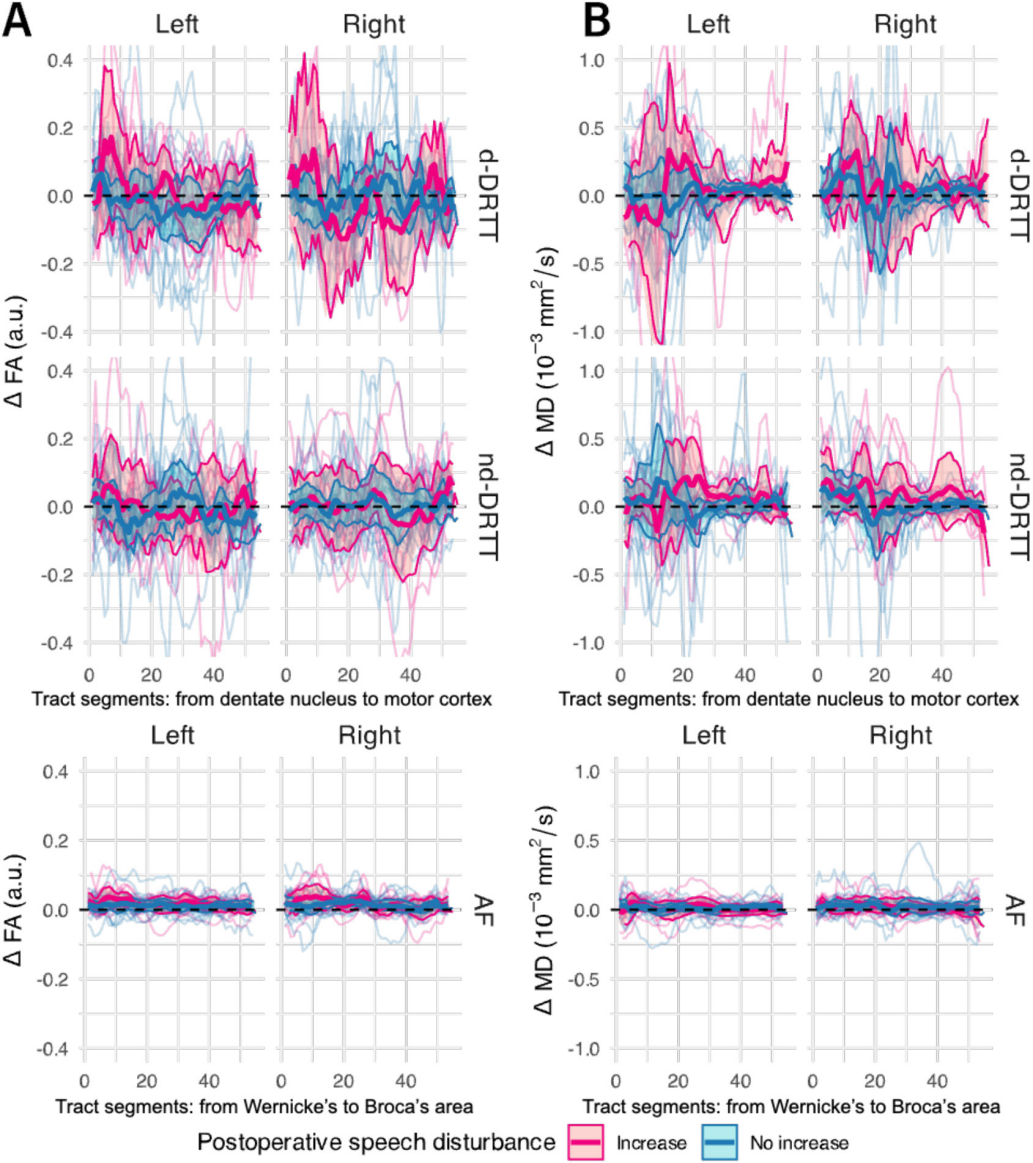
Microstructural changes along the tracts stratified by postoperative speech disturbances. Fractional anisotropy (FA; A) changes between intraoperative and preoperative measurements along the decussating dentato‐rubro‐thalamic tract (d‐DRTT), non‐decussating DRTT (nd‐DRTT), and arcuate fasciculus (AF). Patients are divided into groups according to an increase (pink) or no increase (blue) in postoperative speech disturbance symptoms. Changes in mean diffusivity (MD; B) are shown for the DRTT and AF. “Left” and “Right” indicate the cerebellar hemisphere of the dentate nucleus from which each DRTT originates. Thick lines represent group means, shaded ribbons indicate 95% confidence intervals, thin lines show individual patient trajectories, and the dashed line marks Δ = 0. No significant differences (*p* > 0.05, Wilcoxon rank‐sum test) were observed between groups.

To examine whether microstructural differences along the tracts were related to postoperative speech disturbances, measurements were compared between pPFT patients with and without increased symptoms. Figure 8A displays greater variability in FA along the trajectories of the d‐DRTT and nd‐DRTT in the increased‐symptom group. In comparison, both groups showed similarly little variance in ΔFA along the AF. No significant differences were found between groups in any segment of the tracts (*p* > 0.05). The absolute maximum ΔFA showed the largest group differences in the d‐DRTT (left: 0.13, right: 0.03), followed by the nd‐DRTT (left: 0.03, right: 0.08), and the smallest differences were observed in the AF (left: 0.01, right: 0.01).

Differences between the postoperative speech disturbance groups along the tracts were more evident for MD. Figure 8B shows that children with increased symptoms of speech disturbances exhibited greater variability in MD within the left d‐DRTT, particularly in the segment closer to the dentate nucleus. Children without an increase in symptoms demonstrated relatively stable MD values along the tract. This effect was less pronounced in the nd‐DRTT, while both groups showed minimal differences in ΔFA along the AF. No significant differences were found between groups in any segment of the tracts (*p* > 0.05). The absolute maximum ΔMD (scaled to 10^3^) showed the largest group differences in the nd‐DRTT (left: 1.29, right: 0.15), followed by the d‐DRTT (left: 1.05, right: 0.97), with the smallest differences observed in the AF (left: 0.01, right: 0.25).

## 4 Discussion

We investigated the effect of pPFT surgery on the DRTT by analyzing macro‐ and microstructural changes between preoperative and intraoperative measurements as compared with the AF. We found significant reductions in tract volume and diameter for both d‐DRTT and AF between pre‐ and intraoperative measurements. Reduction of the right d‐DRTT tract volume was negatively correlated with larger ventricular volumes. Whole‐group along‐tract analysis showed high FA variability in the DRTT compared with the AF, and greater MD variability in the cerebellar segments of the DRTT, with larger absolute maximum ΔMD in DRTT components than in the AF. Patients with increased postoperative speech disturbance showed greater FA and MD variability in the DRTT, particularly for MD in the left cerebellar segment.

As hypothesized, the intraoperative reduction of macrostructural properties was more pronounced in the DRTT than in the AF, suggesting a stronger impact of the pPFT surgery on the DRTT, located near the resection cavity. Reduced tract volumes and diameters may reflect local brain collapse after tumor removal, an effect that would be expected to be strongest in the region closest to the lesion, as observed for the DRTT. These findings align with the intraoperative brain shift being more prominent near the resection cavity [17]. In contrast, changes observed in the AF may reflect global intraoperative effects, potentially driven by CSF evacuation [19] and gravity‐induced alterations in brain consistency in the prone position [57, 58]. The proximity of the AF to the ventricles, which are particularly susceptible to CSF leakage, may further contribute to its intraoperative displacement [17, 57]. Although tumor removal may allow local tissue re‐expansion that would theoretically increase tract volumes, the accompanying peritumoral edema introduces isotropic diffusion and greater tract displacement [19], both of which complicate streamline reconstruction and counteract this effect. Consequently, the observed decreases in tract volume and diameter are more likely to reflect intraoperative reconstruction difficulties than actual loss of fibers [59]. Finally, the relatively large voxel size used in this study captures compression and expansion effects more roughly, whereas higher‐resolution imaging could provide a more precise assessment of macrostructural tract changes.

We found that a stronger decrease in tract volume for the right d‐DRTT was significantly associated with larger ventricular volumes. This likely reflects the greater brain shift caused by larger ventricles and tumors [19], which can disrupt the alignment of anatomical regions used in tractography. Such misalignment may lead to the exclusion of streamlines that exceed angle thresholds, thereby reducing tract reconstruction accuracy [60]. Contrary to our hypothesis, no consistent correlations were observed between tumor size and tract volume or diameter. This is in line with evidence that tumor size alone is not a reliable predictor of postoperative outcomes in patients with pPFT [2]. While the resection of larger tumors may increase the risk of damaging eloquent tissue, other factors such as peritumoral edema, tumor consistency, growth pattern, patient age, and the extent of surgical manipulation are likely more decisive than size in determining tract disruption and postoperative neurological complications [2]. These considerations may explain why we observed only a limited relationship between tumor or ventricle size and macrostructural tract properties in our study.

The robustness of our microstructural measurements is supported by the relatively low intra‐patient variance along the trajectory of the AF. This suggests that our measurements are not affected by interscan biases and the observed microstructural effects for the DRTT represent genuine alterations during pPFT surgery. These microstructural alterations were most evident in MD, particularly along the cerebellar segments of the d‐DRTT and nd‐DRTT near the resection cavity, where variability was greater than in their projections to the motor cortex. As hypothesized, the AF showed relatively little along‐tract MD variation, as it lies further from the pPFT surgical site. Consistent with this pattern, whole‐tract MD increased to a greater extent in the nd‐DRTT than in the AF. Although no consistent directional changes in MD were observed across tracts, the magnitude of group‐averaged ΔMD along the tract was larger in both the d‐DRTT and nd‐DRTT than in the AF. This variability in MD may reflect changes in extracellular space or tissue water content, potentially arising from edema, inflammation, or disruption of structural barriers, among others [25, 26]. For FA, the observations differed from those for MD. Along the AF, many segments reached a small but significant intraoperative increase in FA. By comparison, the DRTTs showed larger ΔFA values than the AF, but these changes were scattered across the tract and accompanied by wider confidence intervals, indicating lower precision of FA measures for DRTT reconstruction. At the whole‐tract level, FA increased significantly in the AF, whereas the DRTTs showed only small, non‐significant decreases with higher inter‐patient variability. The overall FA increase in the AF may reflect a reduced partial voluming effect caused by CSF leakage, which can artificially elevate anisotropy [61].

Postoperative speech disturbances were assessed as a proxy for the potentially impaired functionality of the DRTT after pPFT surgery, while acknowledging that the pathophysiology of CMS is far more complex than speech disturbance only. The association between microstructural changes and postoperative speech disturbance symptoms in patients with pPFT was modest in this study. The elevated MD variability observed in the left d‐DRTT of pPFT patients with increased symptoms overlapped with the cerebellar segment that also showed the strongest group‐level sensitivity to surgical manipulation. This overlap could indicate that localized microstructural variability in the cerebellar segment of the DRTT is more vulnerable to postoperative change, potentially related to speech disturbance [2, 62], even if the effects remain below statistical thresholds in this small patient group. In contrast, the AF showed little variability between groups, consistent with its overall stability at the whole‐group level. Additionally, the SARA scale used to retrospectively evaluate postoperative speech disturbances in patients with pPFT in our study does not yet provide a robust assessment of DRTT‐related functional changes. More refined, comprehensive, and prospective approaches are needed to assess how pPFT surgery might affect information integration in the dentate nucleus. This could, for example, be done by assessing motor planning and emotional processing alongside speech control [5, 6, 7, 8, 9].

This study has some limitations. First, a portion of the variance in macrostructural metrics between both acquisitions could be attributed to a reduction in SNR during the intraoperative acquisition. Given that hardware and patient positioning remained constant, this reduction in SNR is likely due to suboptimal coil placement on sterile cloths. This placement increases the distance between the coil and the brain, thereby causing a reduction in the signal being picked up from the brain. The suboptimal coil placement was also observed in two intraoperative datasets, where the field of view did not cover the superior slices of the brain (Data S1). This limited coverage truncated the cortical projections of the DRTT in these patients, introducing variance in macrostructural metrics and cortical along‐tract segments. Nevertheless, despite these potential confounding effects related to SNR and coverage, the differences in macrostructural metrics remained statistically significant and did not affect the microstructural findings in the cerebellar segments. Furthermore, our study population included children aged 21 months, an age when myelination is not yet complete. Completion of myelination typically varies per white matter tract between 0.49 and 2.1 years of age [63]. Incomplete myelination is generally associated with lower FA and higher MD [63], which could affect microstructural analysis. However, the dentato‐thalamo portion of the DRTT undergoes earlier myelination (before 1.6 years of age) than projection tracts (more than 2 years of age) [63]. Moreover, our analyses relied on within‐patient comparisons rather than absolute values across children, thereby minimizing age‐related effects. Consistently, age was not associated with increased postoperative speech disturbance symptoms. Another limitation is that we were unable to distinguish between tumor types in our analyses due to small subgroup sizes. More generally, it should be also acknowledged that tractography is a computational method that next to real anatomy can reconstruct false positives and negatives [64]. Although we optimized the parameters for intraoperative reconstruction in a previous study [27], intraoperative findings should be interpreted with care in light of known anatomy.

For future research, it would be interesting to improve the quality and robustness of streamline reconstruction at the decussation of the d‐DRTT that often fails due to crossing fibers and sharp turns [65]. One potential solution is to reconstruct the d‐DRTT in two segments, before and after the decussation, and then merge them. This could help to increase the d‐DRTT volume that is expected to be larger than the nd‐DRTT based on dissection evidence [51]. Enhancing SNR of the intraoperative data, gathering prospective clinical data, and improving overall data quality will also be essential to increasing reconstruction reliability and allow more accurate assessment of how macro‐ and microstructural changes relate to functional outcomes, including postoperative speech disturbances. In conclusion, pPFT surgery affected the variability of the DRTT microstructure near the resection cavity, whereas the variability of the AF remained relatively stable. Although we did not find significant differences in patients with postoperative speech disturbances, we observed a much higher variability in MD in this group, suggesting a potential effect of DRTT disruption in these symptoms.

## Author Contributions

All authors contributed to the study conception and design. MRI data acquisition was performed by P. Jellema and E. Hoving. Fiber tractography analysis was performed by P. Jellema and A. De Luca. Regions of interest were segmented by P. Jellema and validated by W. Nieuwenhuis. K. Kersbergen and E. Hoving provided advice on the clinical interpretation of the data. J. Wijnen, M. Froeling, and A. De Luca provided technical advice on the fiber tractography data analysis. The first draft of the manuscript was written by P. Jellema, and all authors commented on previous versions of the manuscript. All authors read and approved the final manuscript.

## Funding

This work was supported by the European Research Council, 101163214. Alzheimer Nederland, WE.03‐2022‐11, Galen and Hilary Weston Foundation, Hanarth Fonds.

## Ethics Statement

The local ethics committee approved this study (BDAC number: PMCLAB2022.0379). All subjects and/or caregivers provided written informed consent.

## Consent

The authors affirm that human research participants provided informed consent for publication of the images in this paper.

## Conflicts of Interest

The authors declare no conflicts of interest.

## Supporting information


**Data S1:** Dentato‐rubro thalamic tract reconstructions.

**Data S2:** Arcuate fasciculus reconstructions.

## Data Availability

The datasets generated and/or analyzed during the current study are not publicly available due to patient privacy, but are available from the corresponding author on reasonable request.
