# Supplementary material for: Structural Fiber Tract Alterations in Relation to Surgery in Children With a Posterior Fossa Tumor
Source: NMR Biomed. 2026 Feb 20;39(4):e70250. doi: 10.1002/nbm.70250 (PMC12923661; doi:10.1002/nbm.70250)
Supplement: Supplementary file 1 — Data S1: Dentato‐rubro thalamic tract reconstructions. [file NBM-39-e70250-s002.docx]

**Supplementary material 1.**

**Dentato-rubro thalamic tract reconstructions**

Left (1) or right (2) decussating or non-decussating dentato-rubro-thalamic tract (DRTT)

corresponds to the hemisphere of the dentate nucleus from which the tract originates.


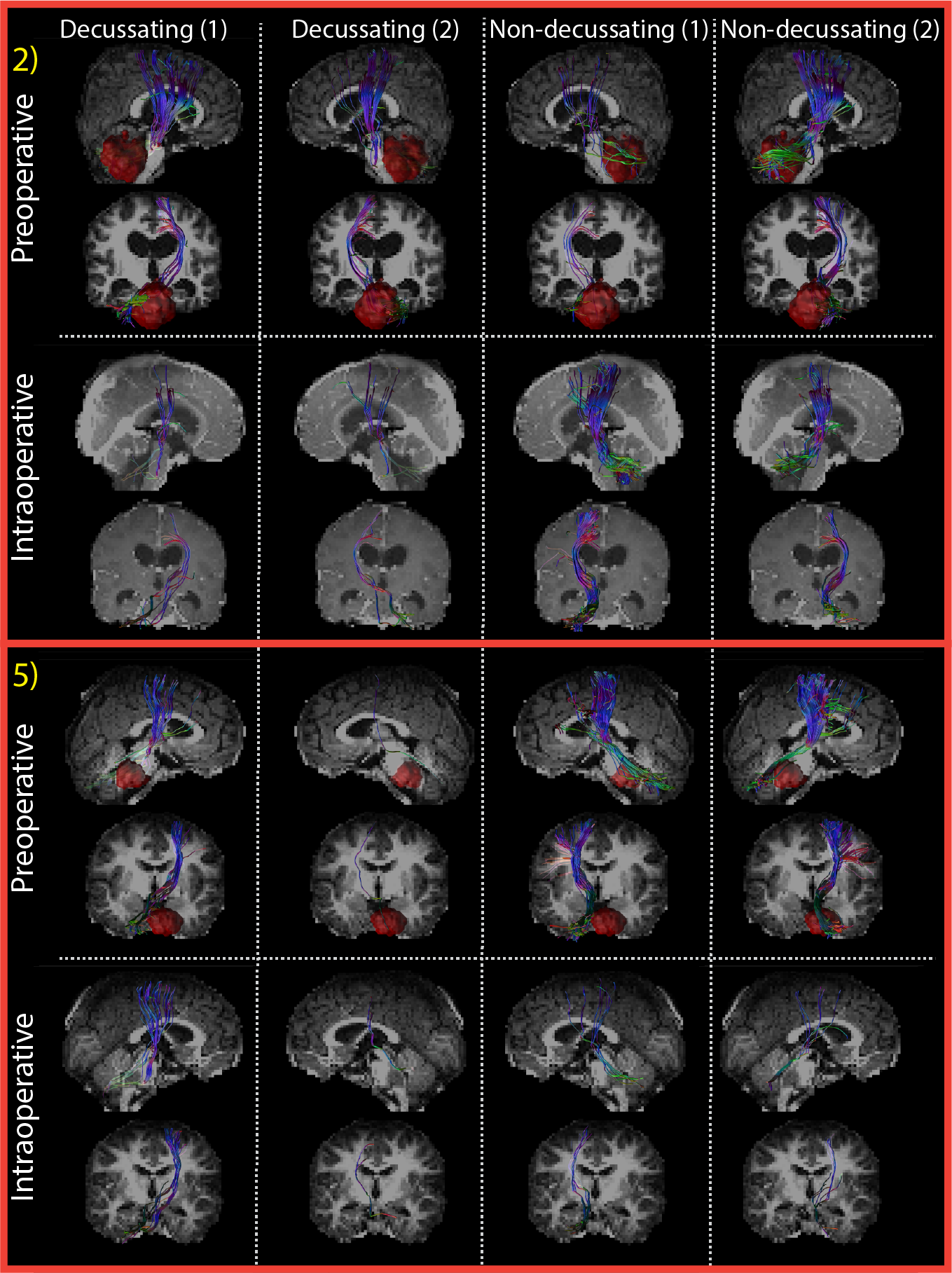


Figure 1. DRTT reconstructions of patients 2 and 5. Note that the T1-weighted image of patient 2 is not an MPRAGE but a 3D multishot turbo field echo.


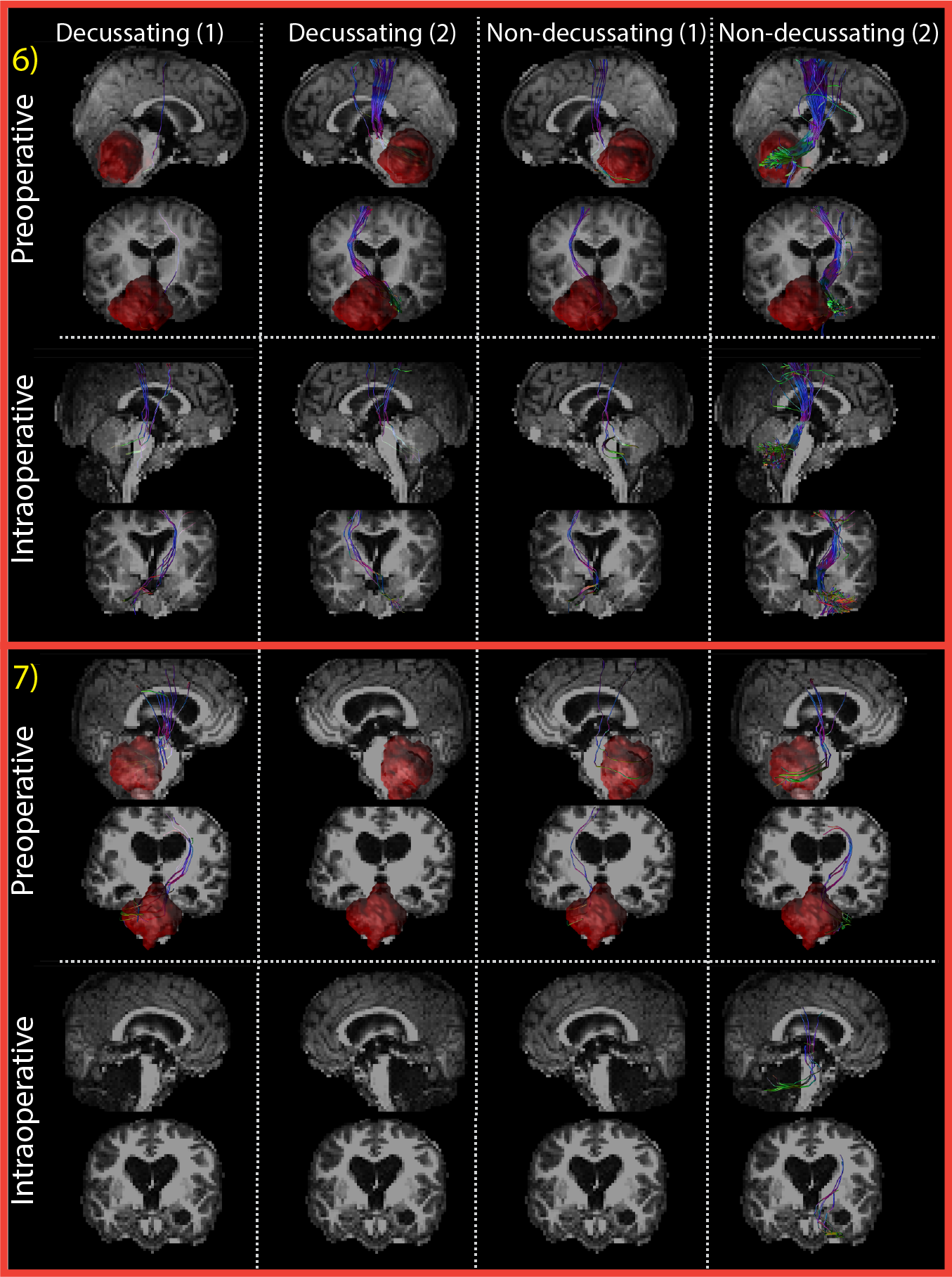


Figure 2. DRTT reconstructions of patients 6 and 7. Note that the field of view of patient 6 is not covering the tip of the brain because of the caudal position of the coil.


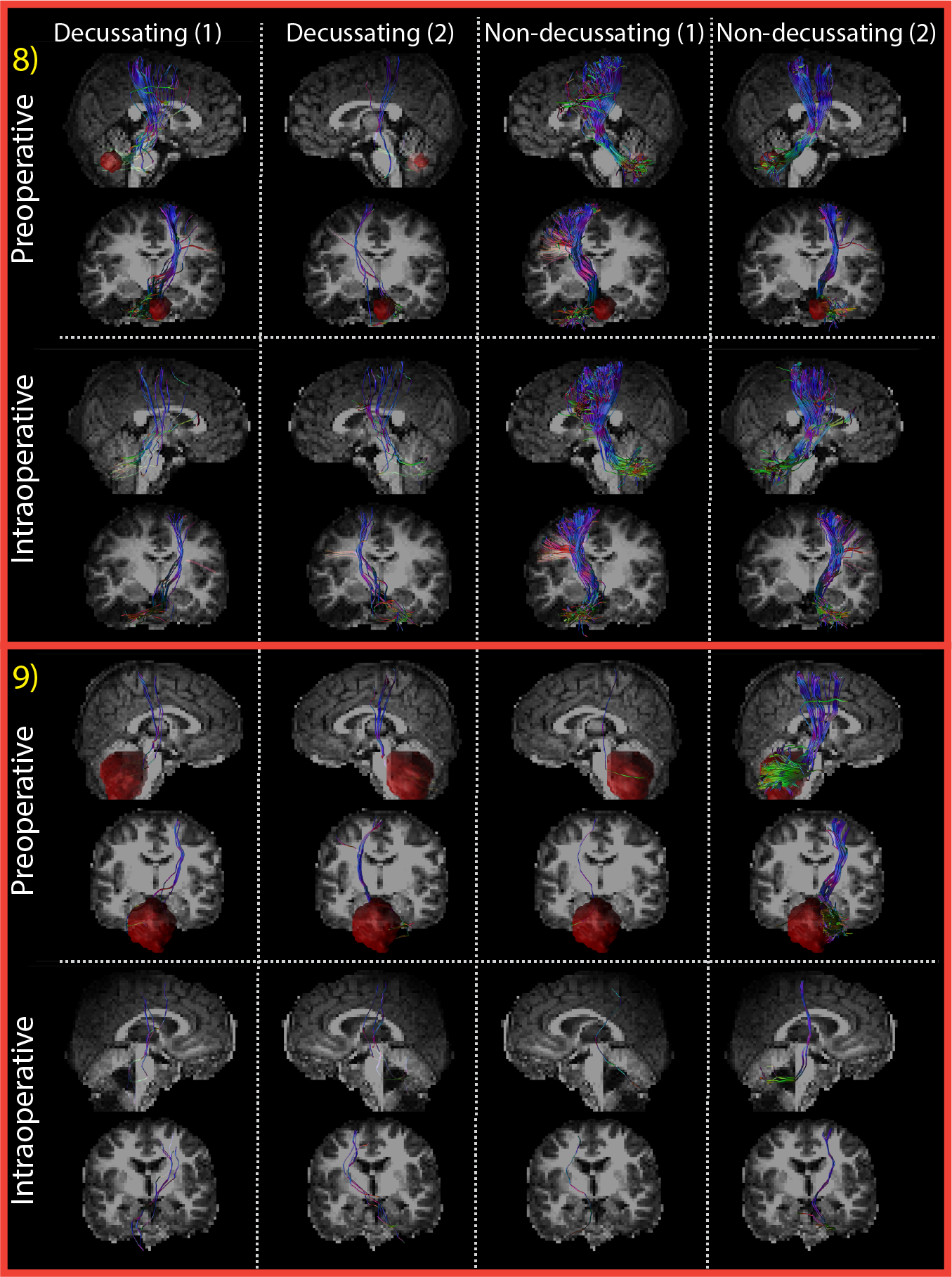


Figure 3. DRTT reconstructions of patients 8 and 9.


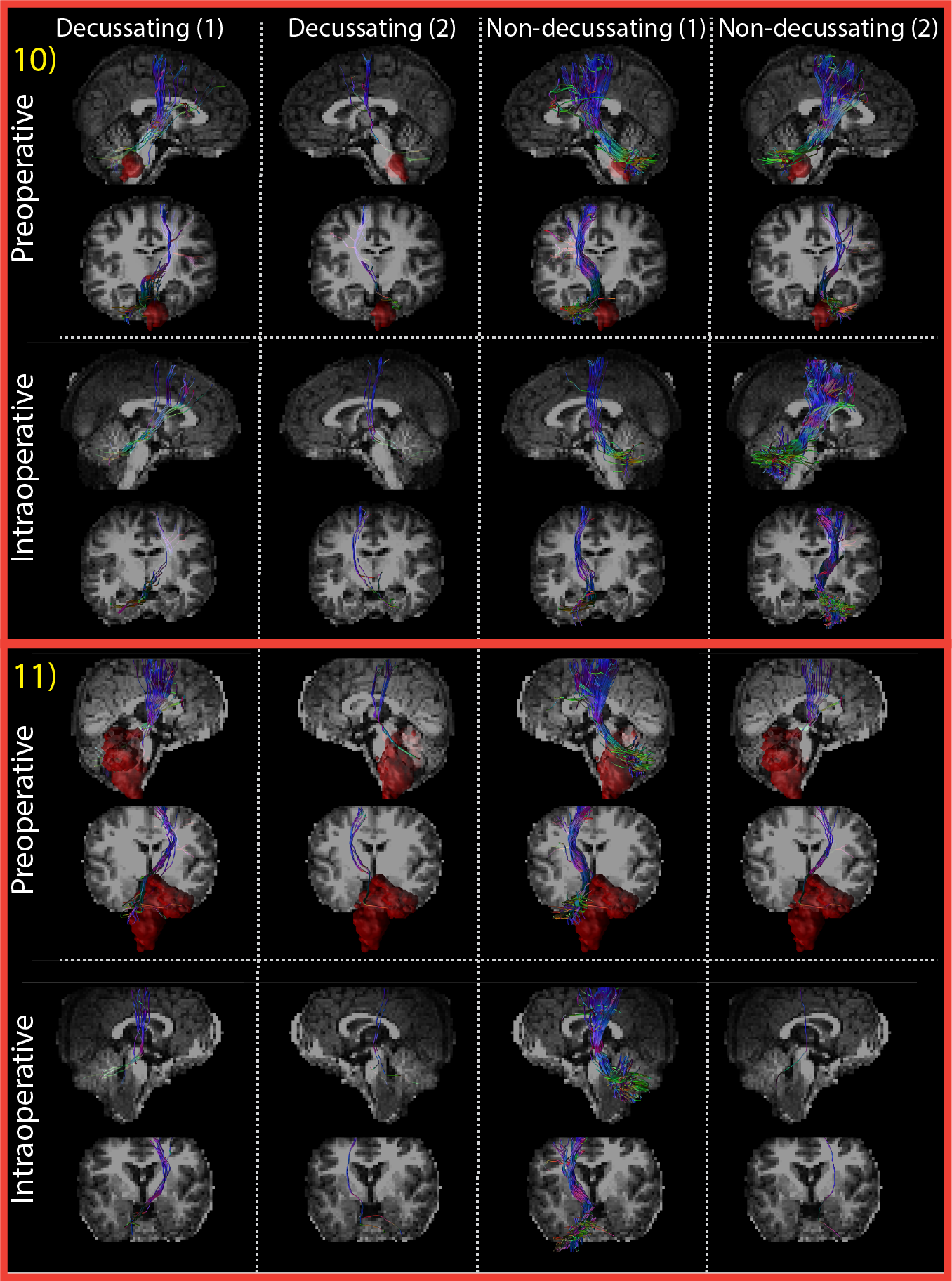


Figure 4. DRTT reconstructions of patients 10 and 11. Note that the field of view of patient 11 is not covering the tip of the brain because of the caudal position of the coil.


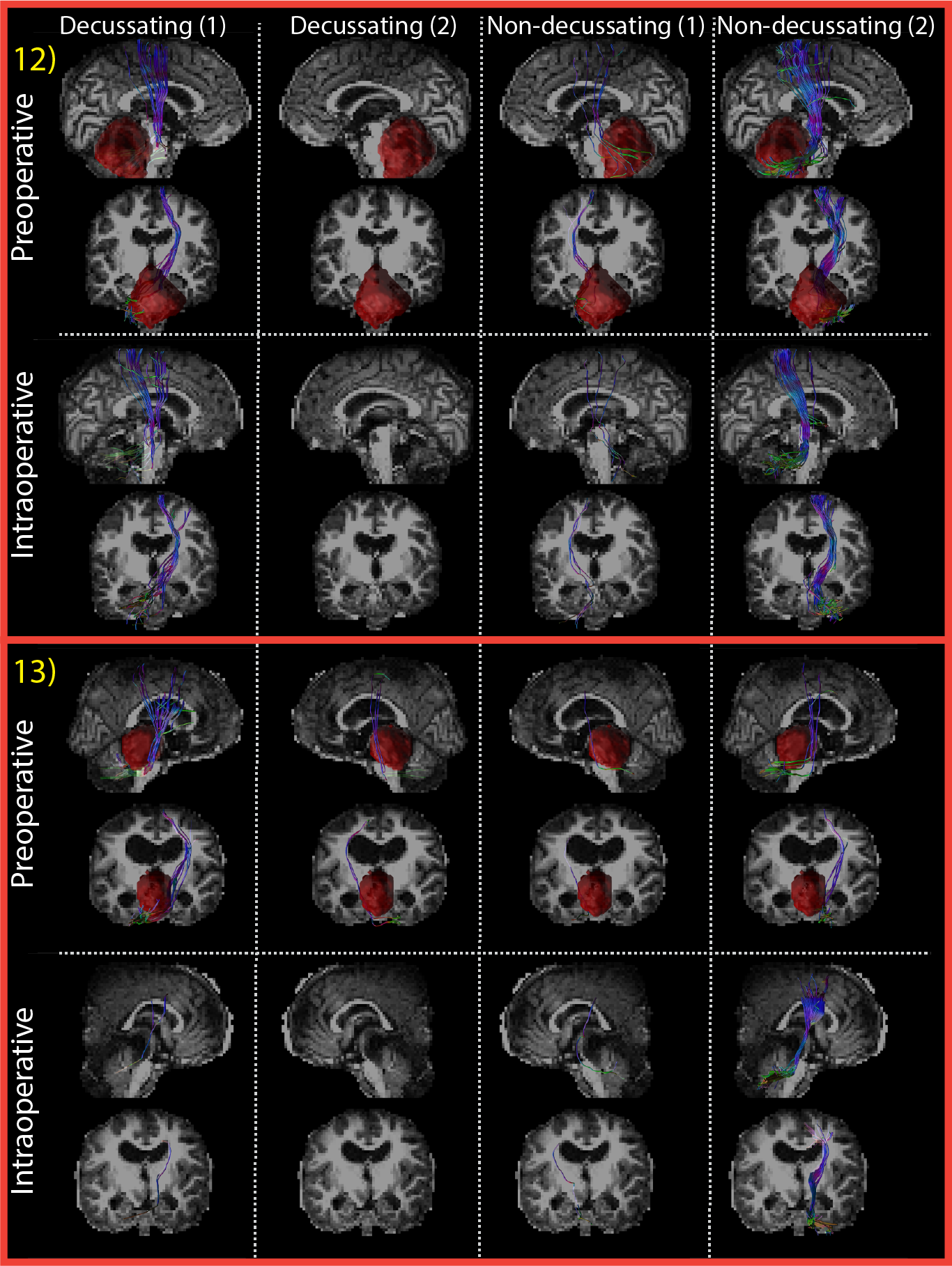


Figure 5. DRTT reconstructions of patients 12 and 13.


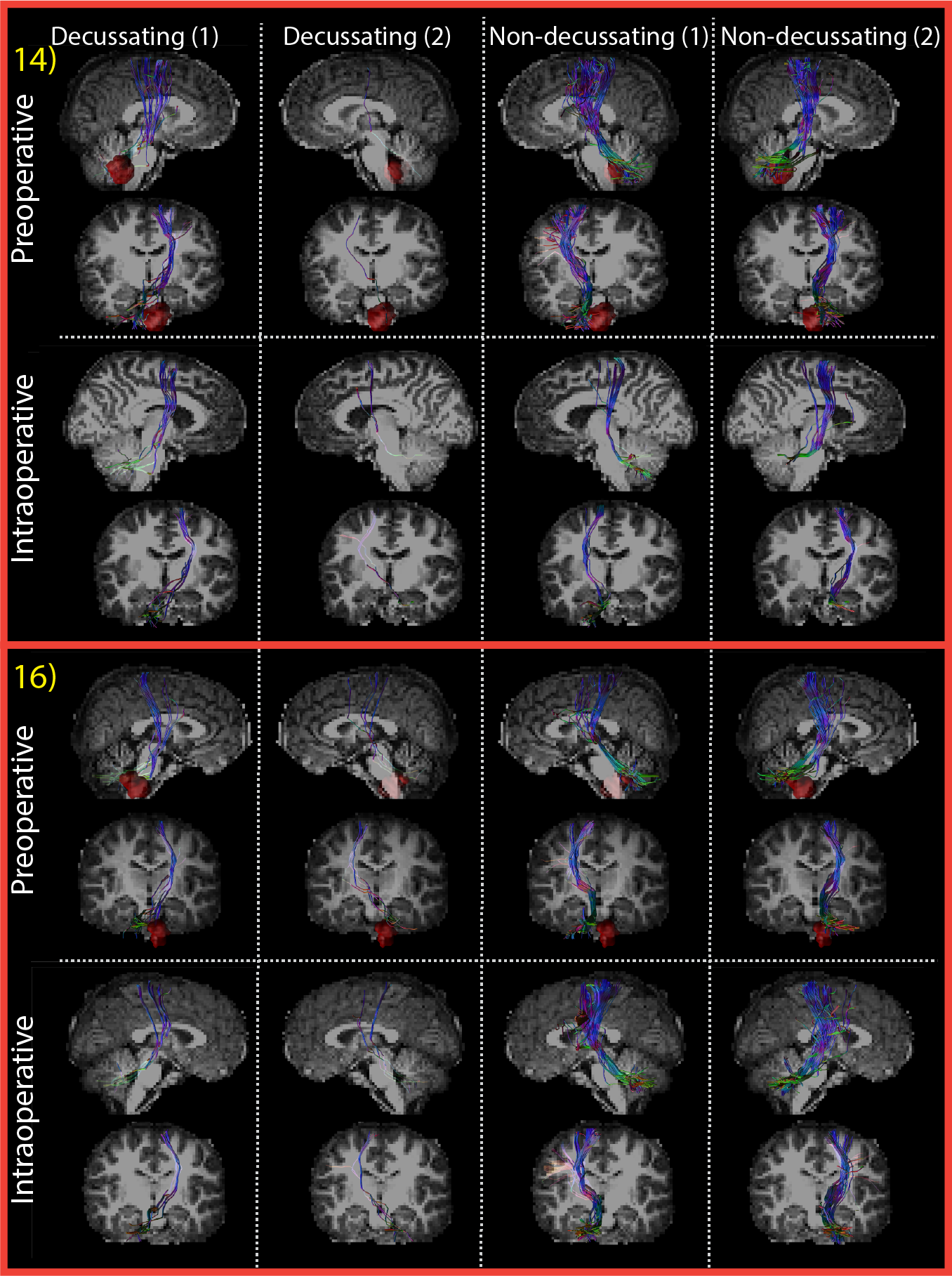


Figure 6. DRTT reconstructions of patients 14 and 16.


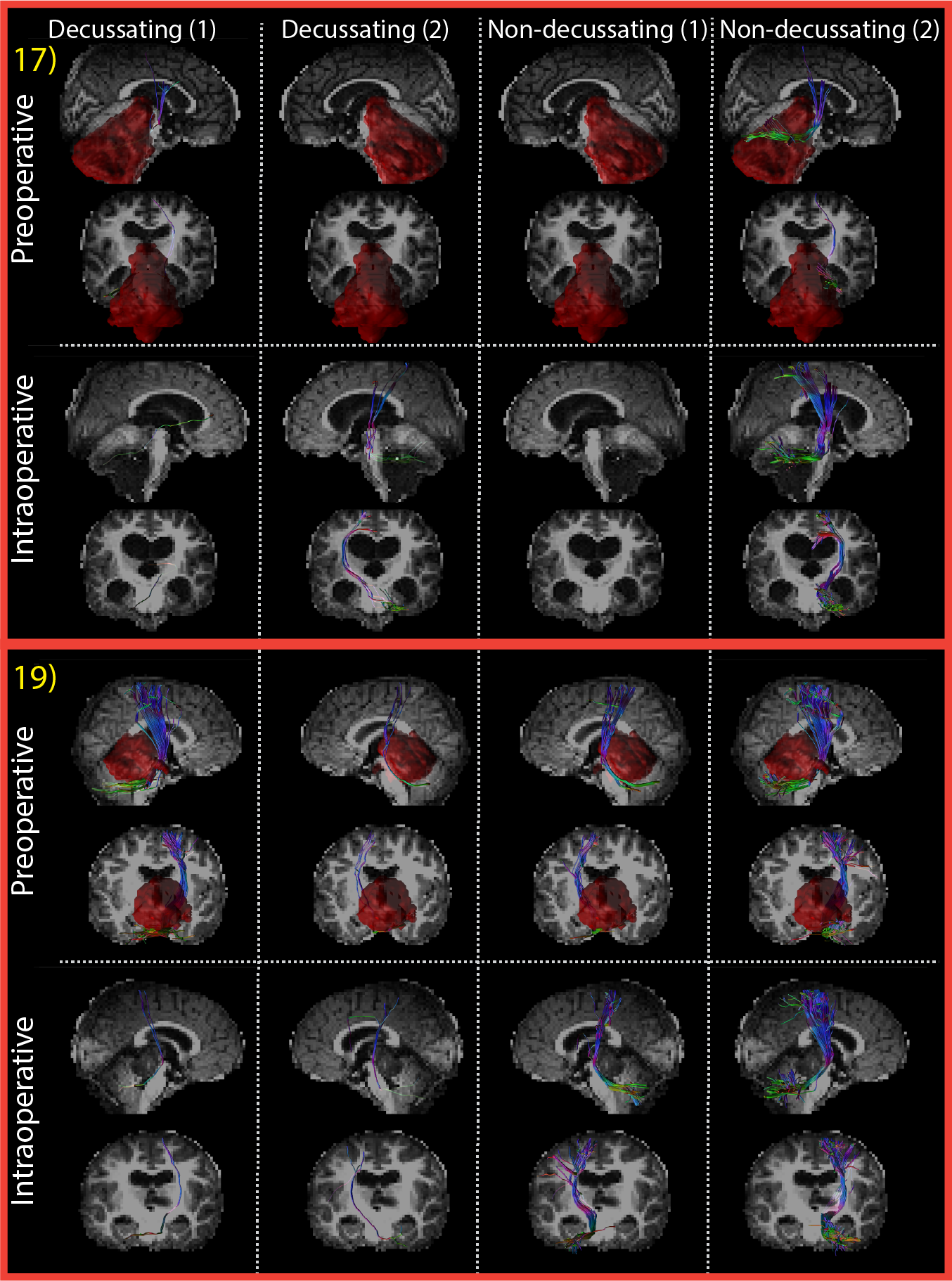


Figure 7. DRTT reconstructions of patients 17 and 19.


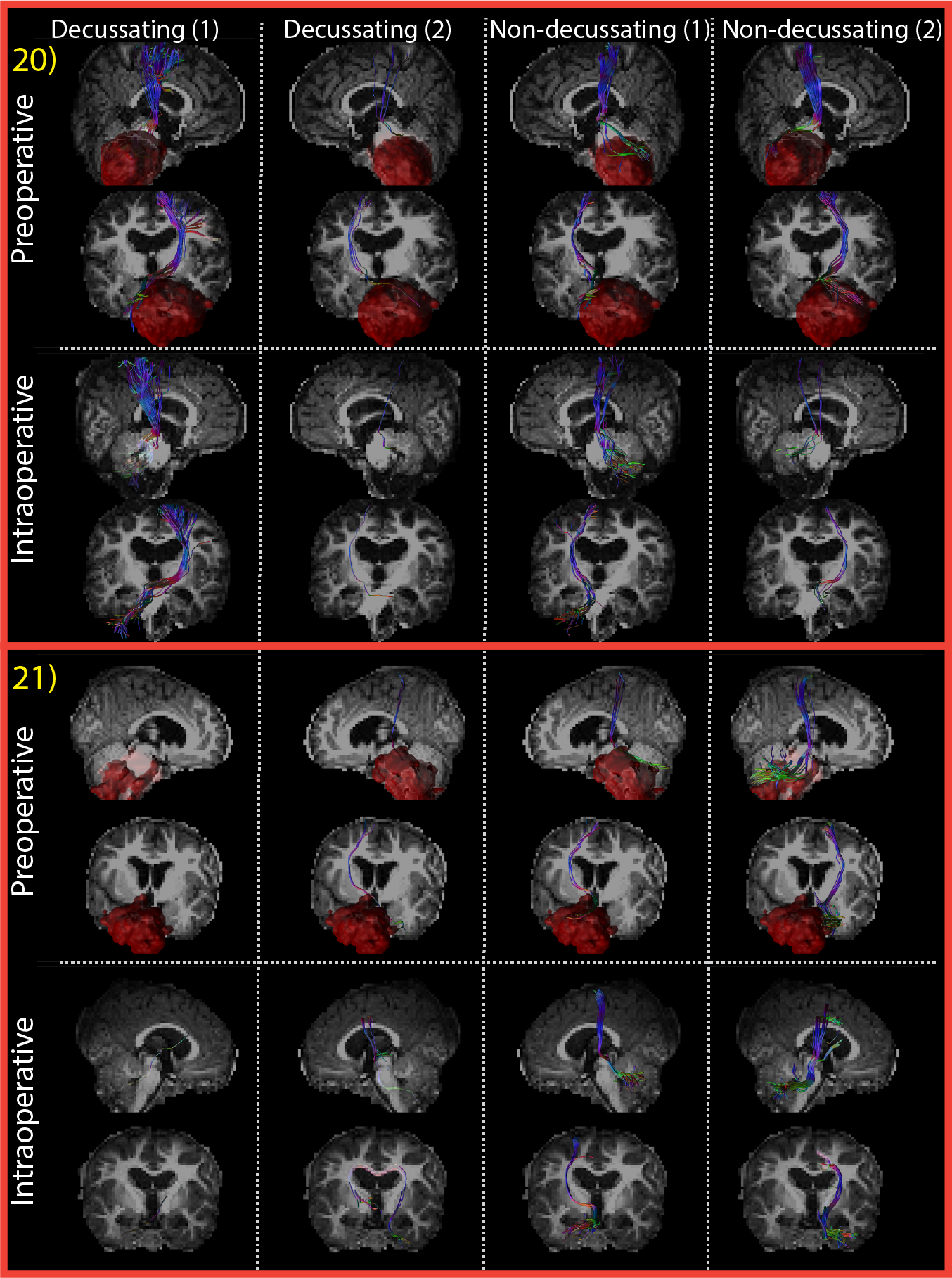


Figure 8. DRTT reconstructions of patients 20 and 21.


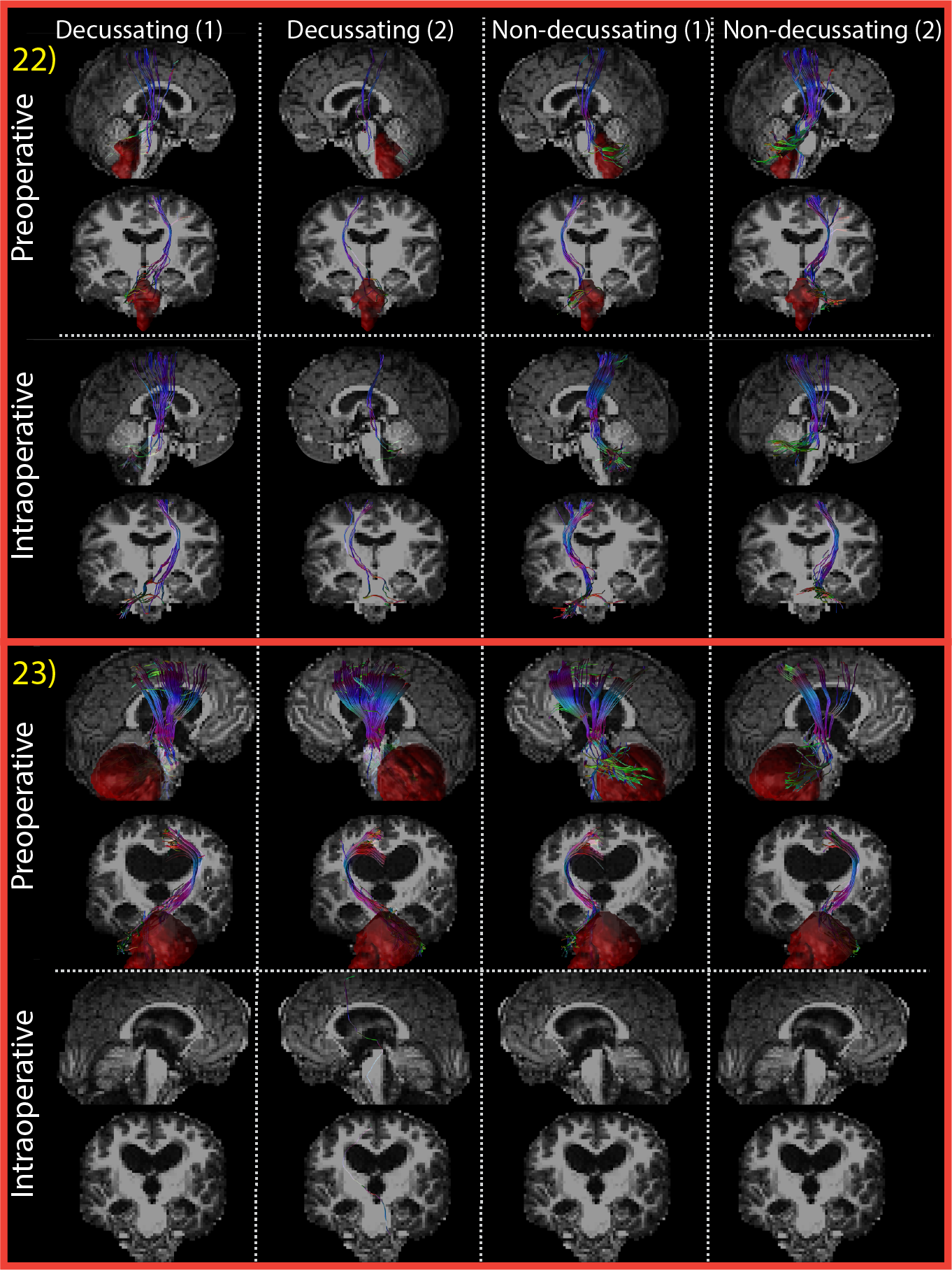


Figure 9. DRTT reconstructions of patients 22 and 23.


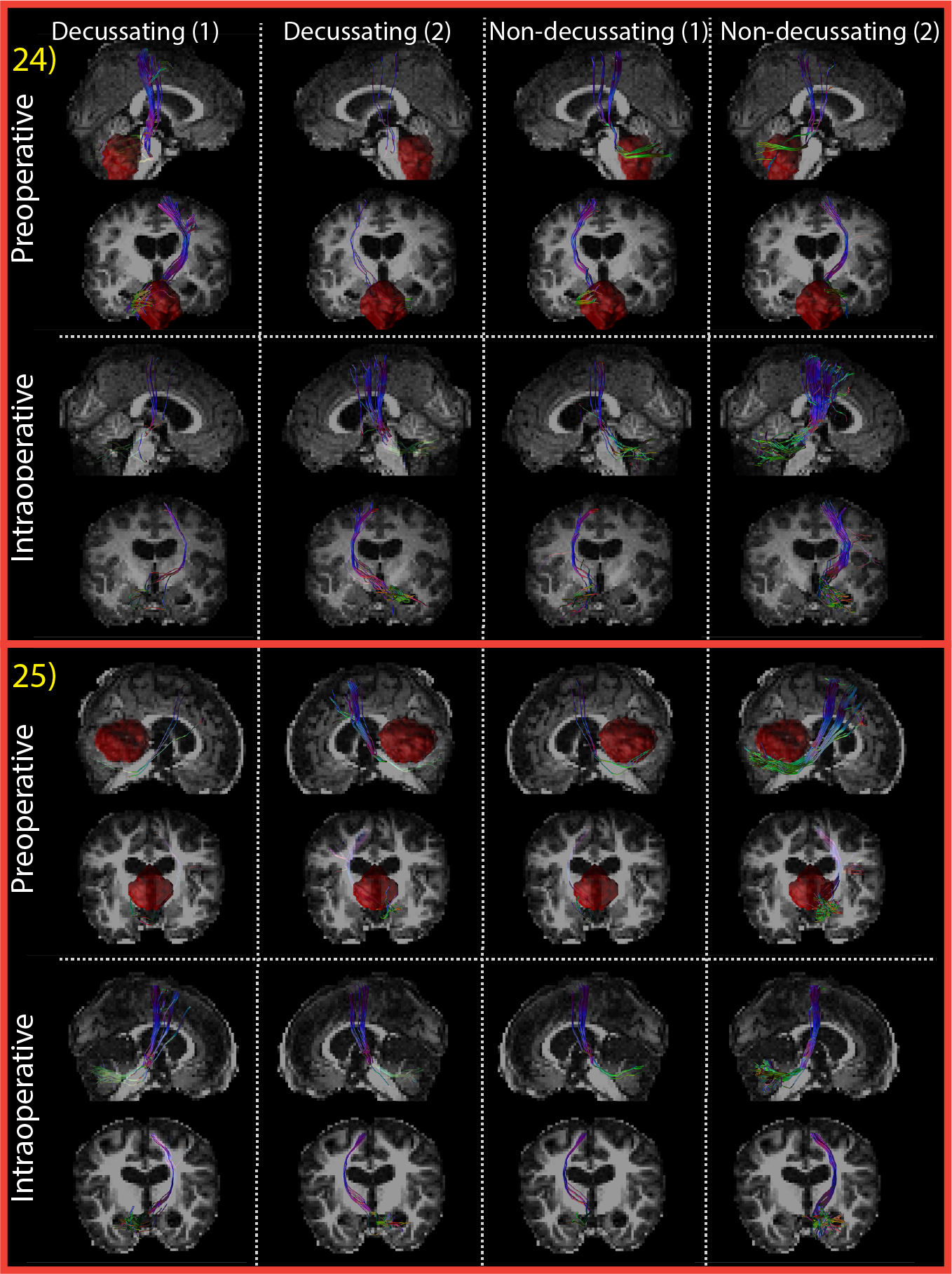


Figure 10. DRTT reconstructions of patients 24 and 25.


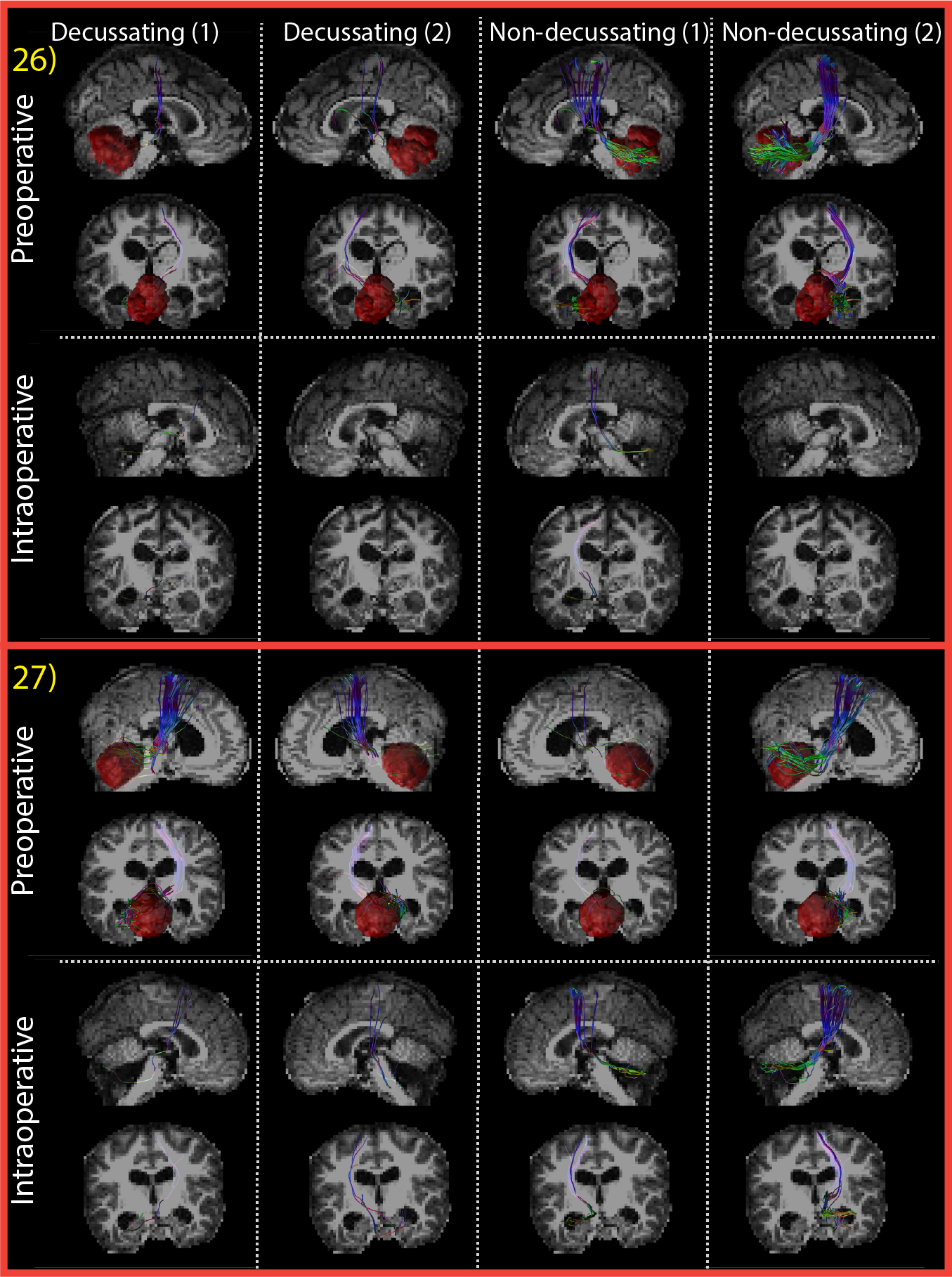


Figure 11. DRTT reconstructions of patients 26 and 27.


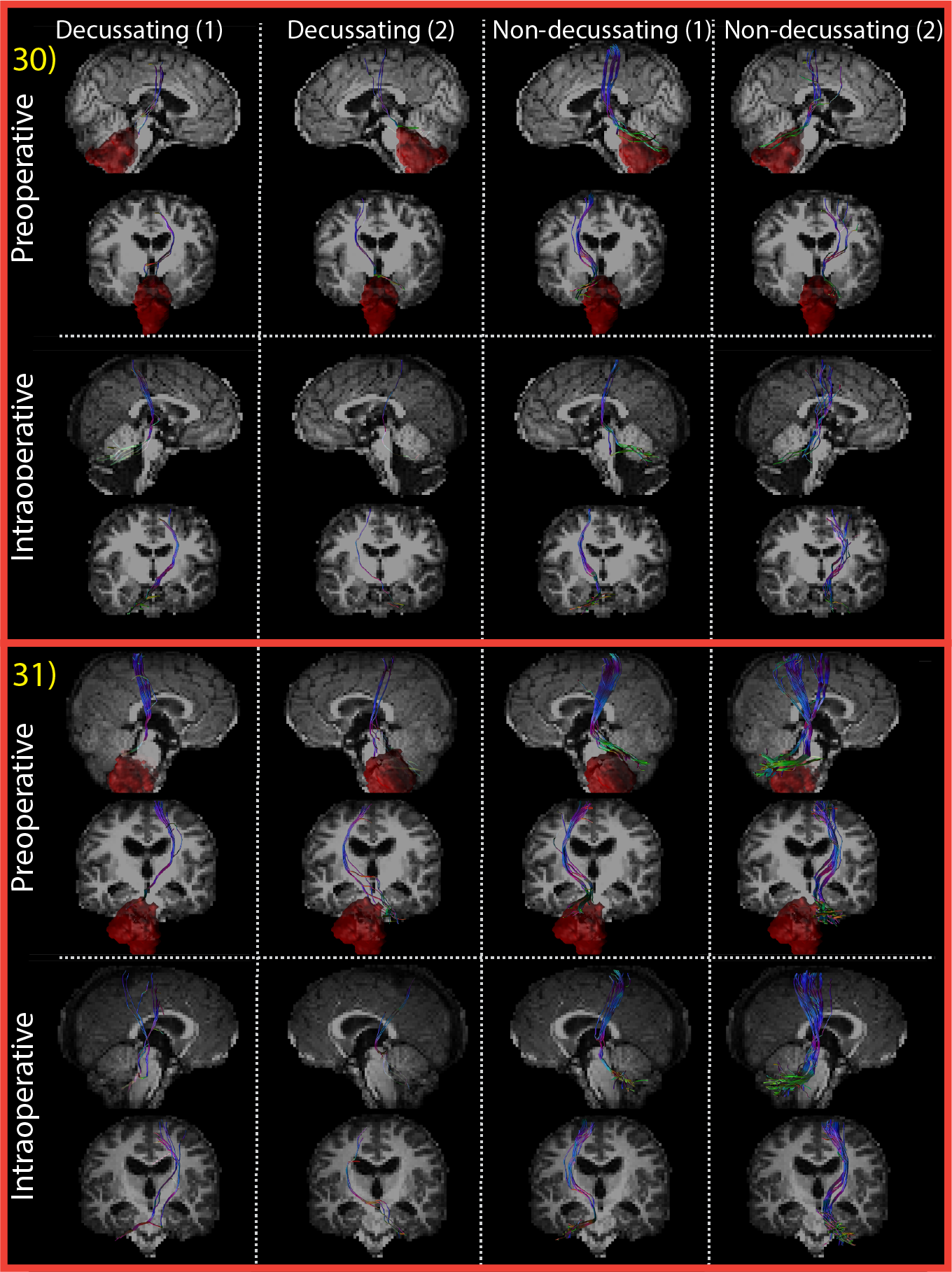


Figure 12. DRTT reconstructions of patients 30 and 31.


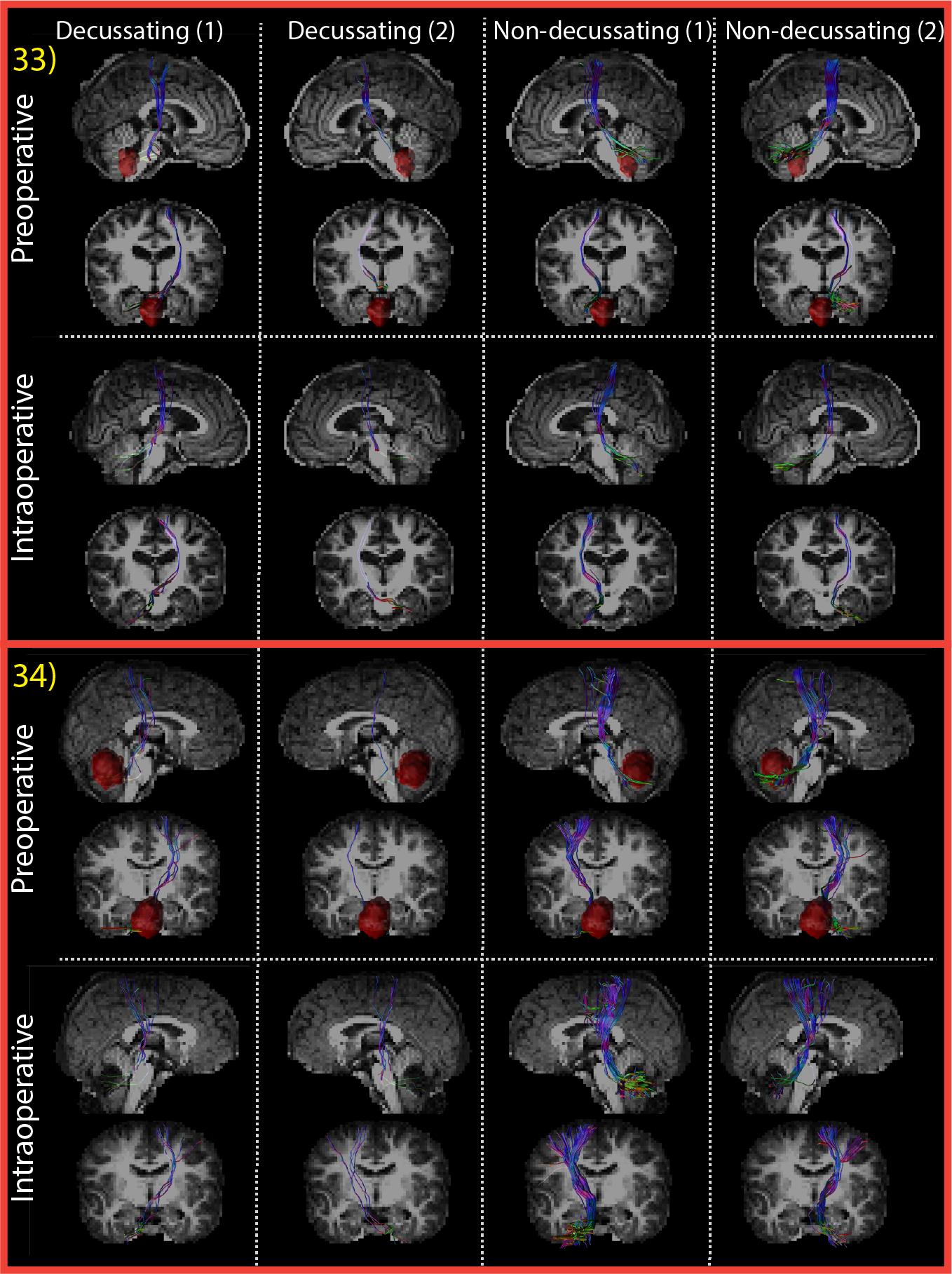


Figure 13. DRTT reconstructions of patients 33 and 34.


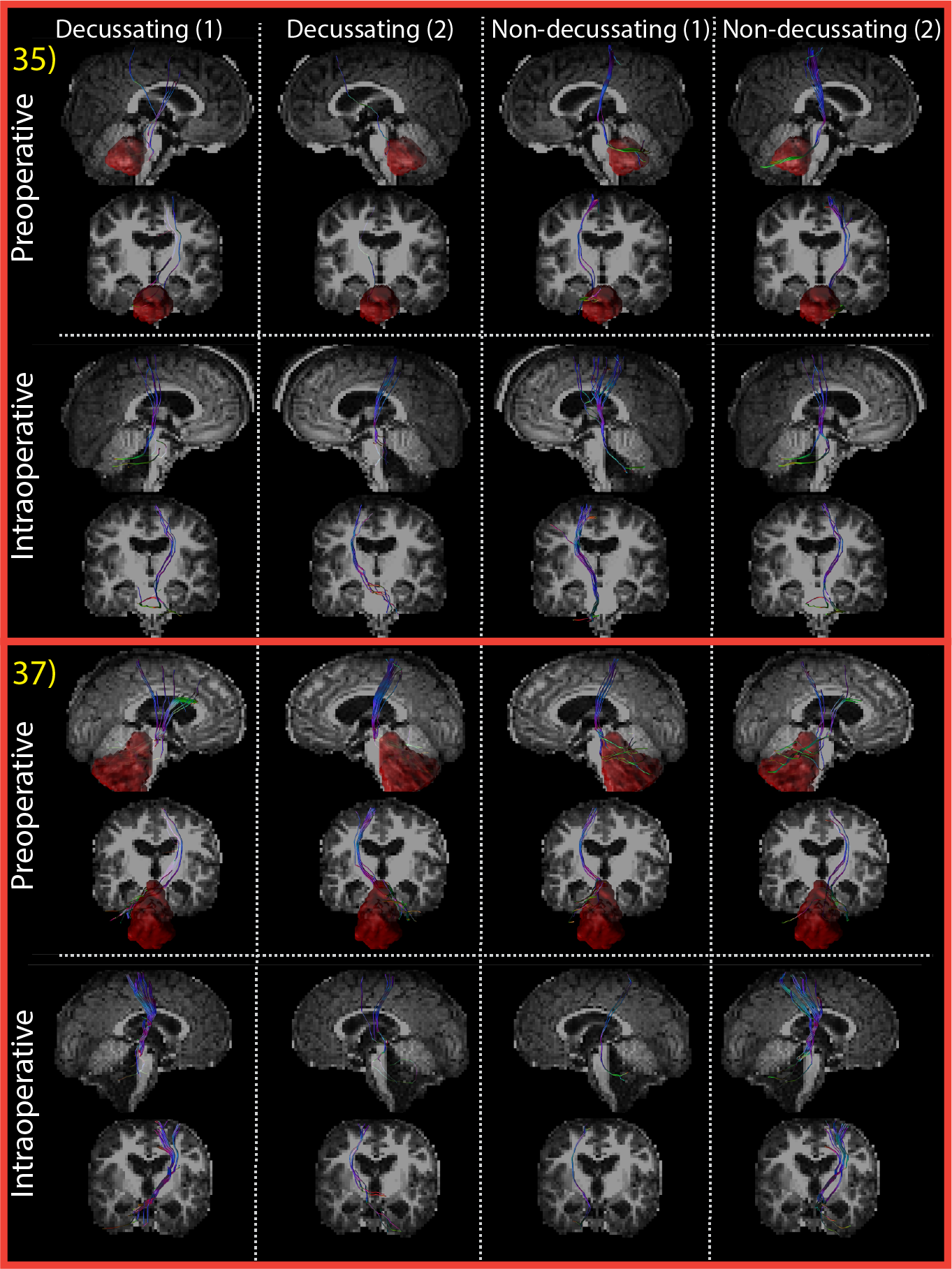


Figure 14. DRTT reconstructions of patients 35 and 37.


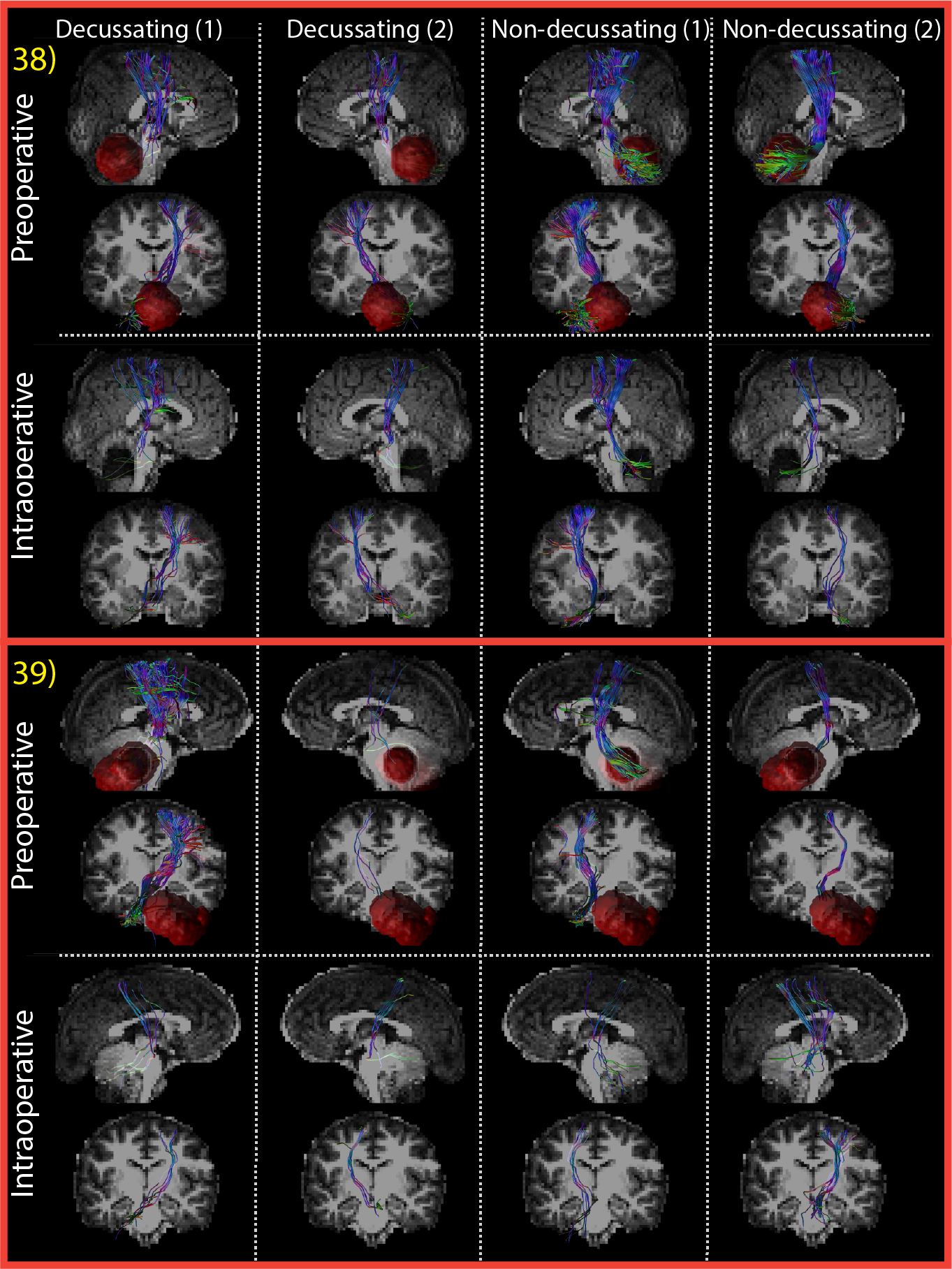


Figure 15. DRTT reconstructions of patients 38 and 39.
