# Supplementary material for: Structural Fiber Tract Alterations in Relation to Surgery in Children With a Posterior Fossa Tumor
Source: NMR Biomed. 2026 Feb 20;39(4):e70250. doi: 10.1002/nbm.70250 (PMC12923661; doi:10.1002/nbm.70250)
Supplement: Supplementary file 2 — Data S2: Arcuate fasciculus reconstructions. [file NBM-39-e70250-s001.docx]

**Supplementary material 2.**

**Arcuate fasciculus reconstructions**

Examples are shown of all patients of the left (1) and right (2) arcuate fasciculus (AF) reconstructions.


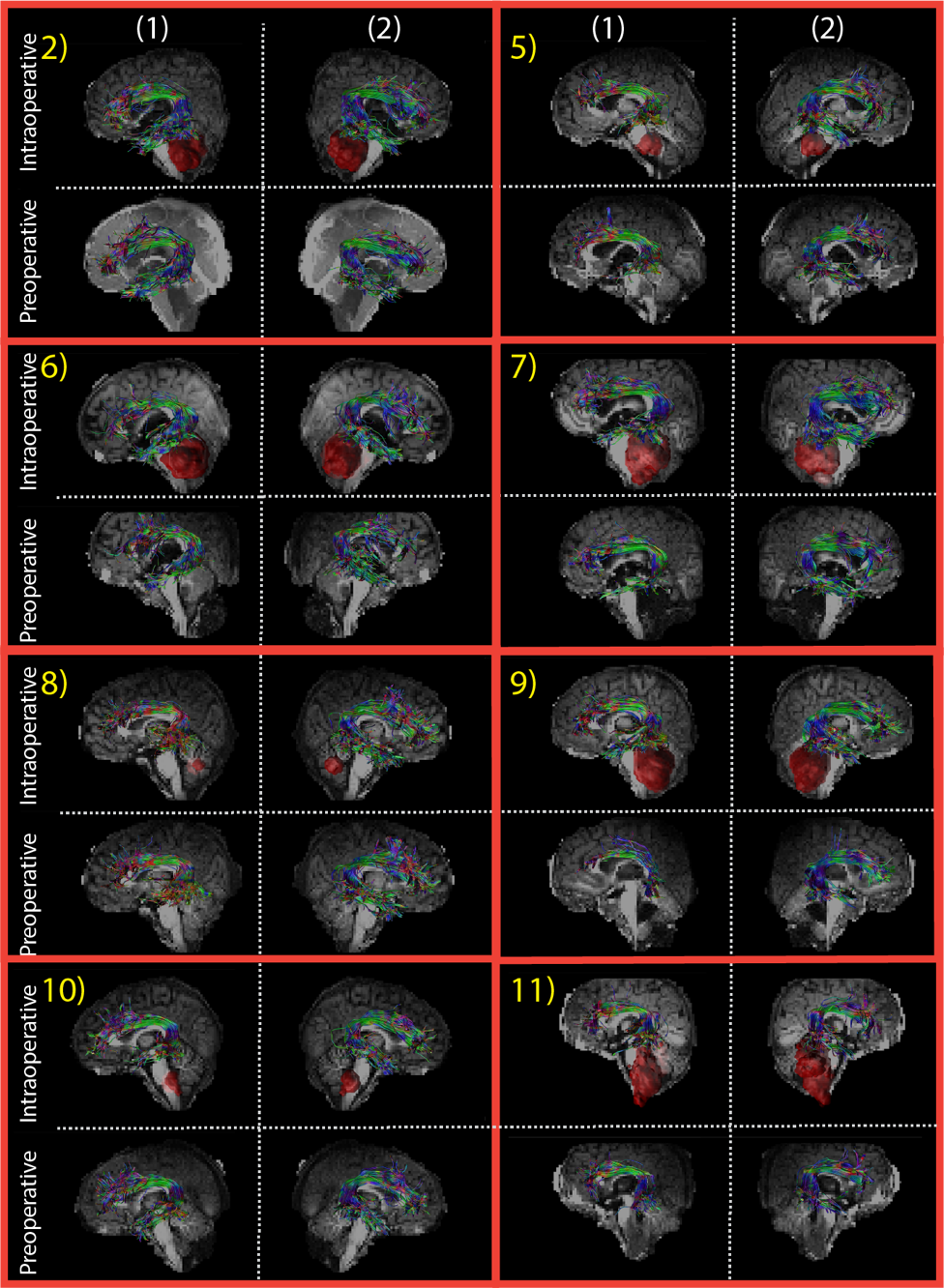


Figure 1. Arcuate fasciculus reconstructions of patients 2 till 11. Note that the T1-weighted image of patient 2 is not an MPRAGE but a 3D multishot turbo field echo. The field of view of patients 6 and 11 are not covering the tip of the brain because of the caudal position of the coil.


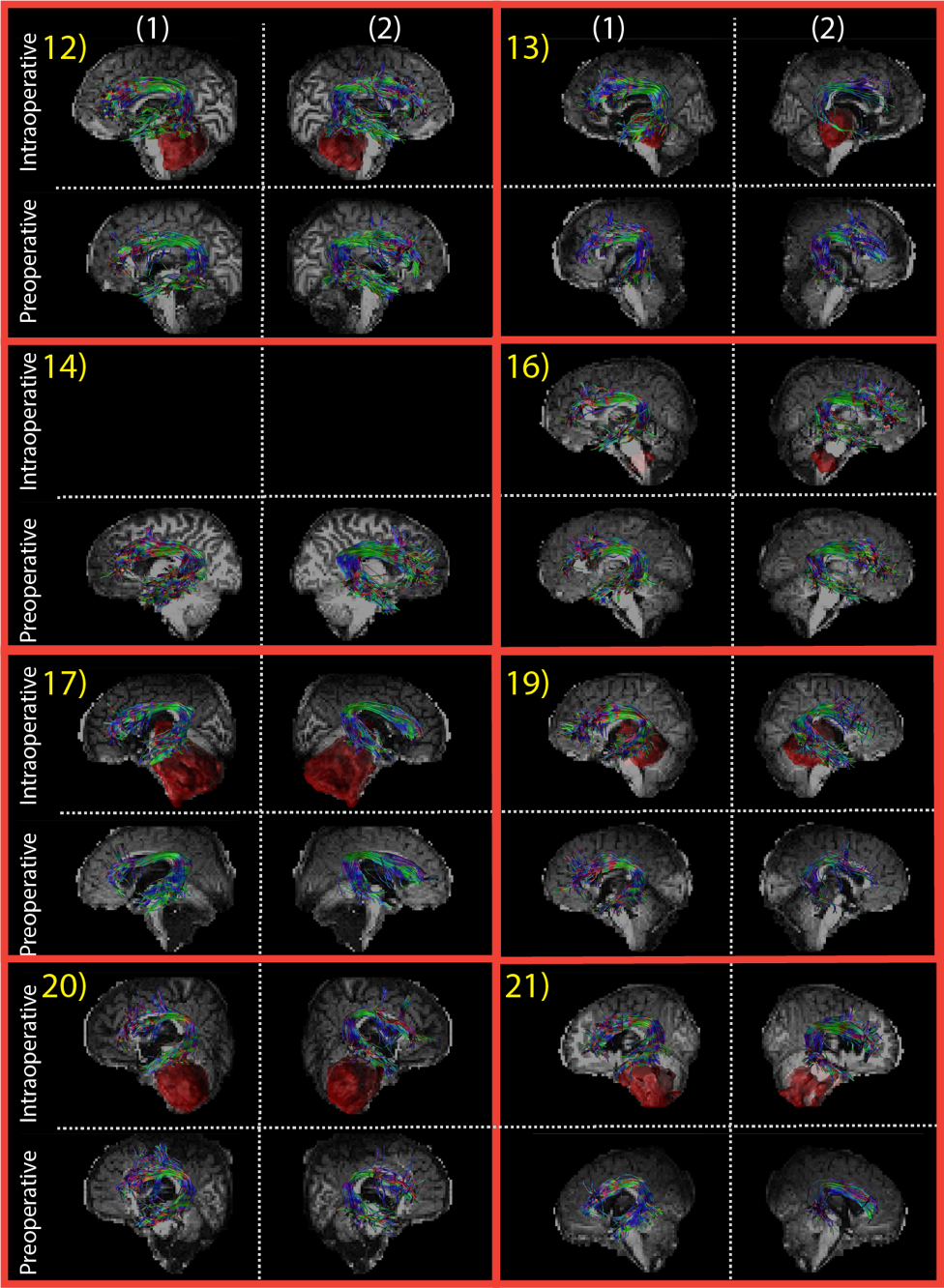


Figure 2. Arcuate fasciculus (AF) reconstructions of patients 12 till 21. Note that the AF failed to reconstruct in the preoperative data of patient 14.


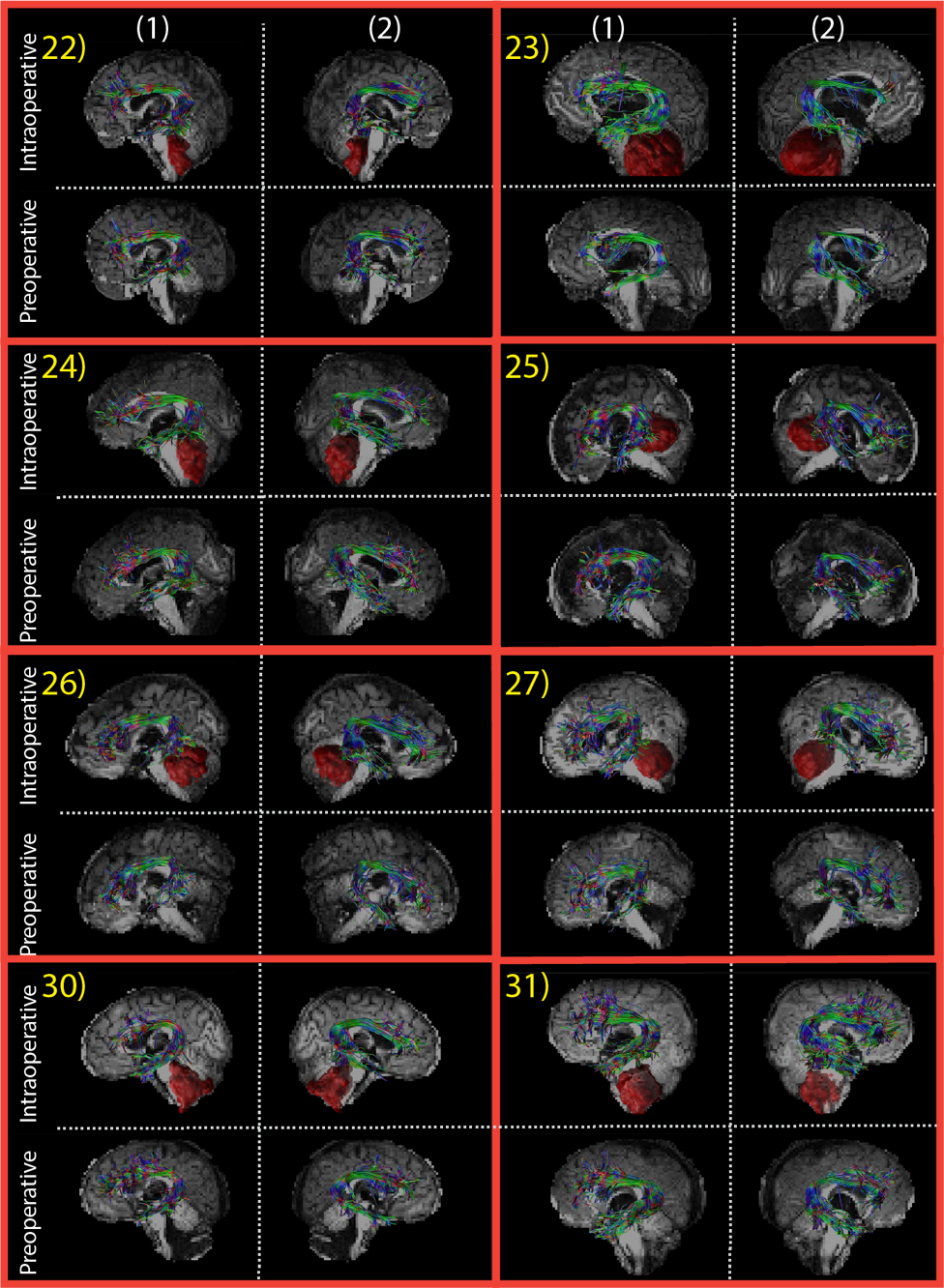


Figure 3. Arcuate fasciculus reconstructions of patients 22 till 31.


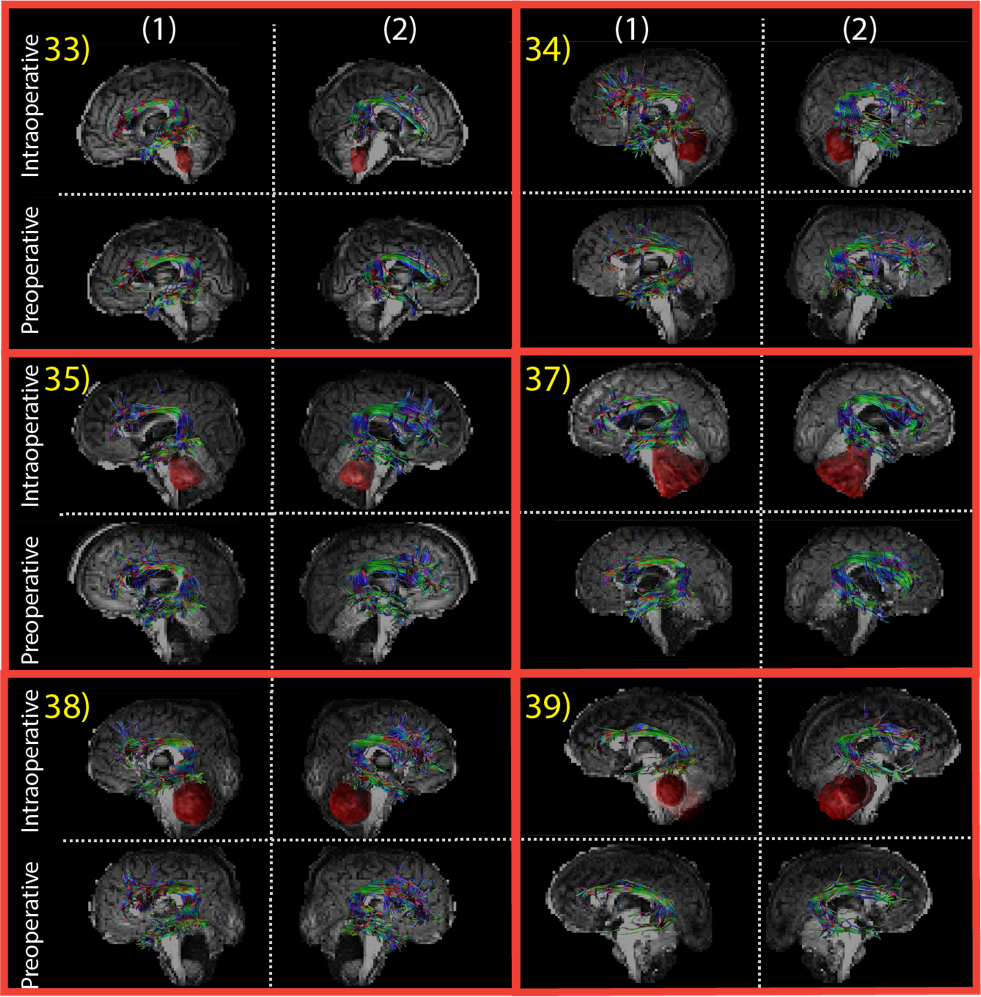


Figure 4. Arcuate fasciculus reconstructions of patients 33 till 39.
